# Supplementary figures and images for: The variable prevalence of bovine tuberculosis among dairy herds in Central Ethiopia provides opportunities for targeted intervention
Source: PLoS One. 2021 Jul 2;16(7):e0254091. doi: 10.1371/journal.pone.0254091 (PMC8253440; doi:10.1371/journal.pone.0254091)

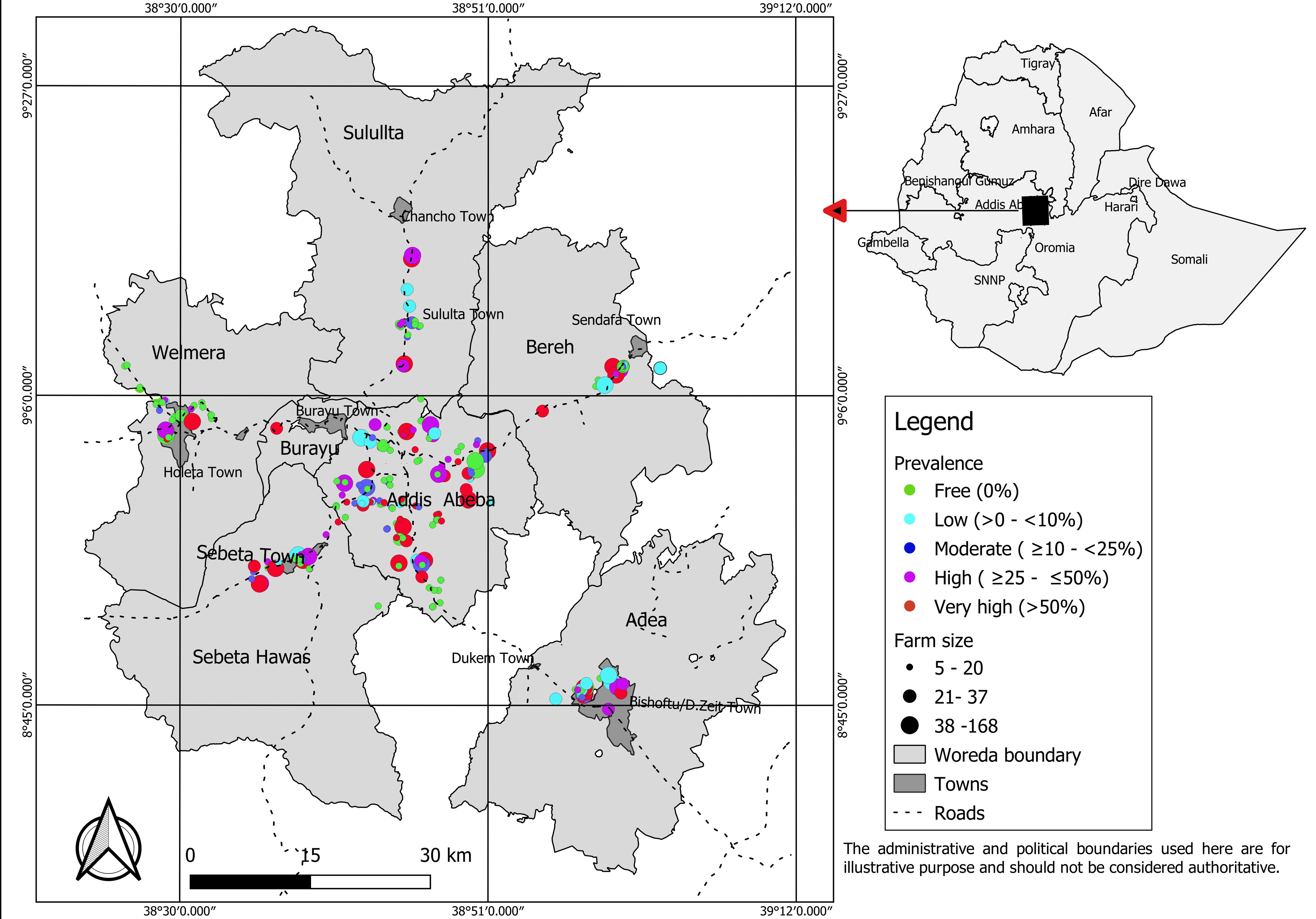

Supplement: S1 Fig — (TIF) [file pone.0254091.s001.tif]
