# Supplementary material for: The variable prevalence of bovine tuberculosis among dairy herds in Central Ethiopia provides opportunities for targeted intervention
Source: PLoS One. 2021 Jul 2;16(7):e0254091. doi: 10.1371/journal.pone.0254091 (PMC8253440; doi:10.1371/journal.pone.0254091)
Supplement: S1 Table — (DOC) [file pone.0254091.s002.doc]

**S1 Table. Characteristics of studied farms**

| **Characteristics** | **levels** | **Small farm (n=212)** | **Medium farm (n=49)** | **Large farm (n=38)** | **Total**  **(n=299)** |
| --- | --- | --- | --- | --- | --- |
| Farm ownership | Private | 171 | 37 | 30 | 238 |
|  | Government | 5 | 1 | 2 | 8 |
|  | Cooperatives | 27 | 4 | 0 | 31 |
|  | Share company | 4 | 3 | 3 | 10 |
|  | Missed values/data not collected | 5 | 4 | 3 | 12 |
| Manure disposal method | Used as organic fertilizer/fuel | 34 | 12 | 7 | 53 |
|  | Disposed/accumulated in the farm compound | 117 | 21 | 15 | 153 |
|  | Open pit | 44 | 11 | 11 | 66 |
|  | Septic tank | 11 | 3 | 5 | 19 |
|  | Missed values/data not collected | 6 | 2 | 0 | 8 |
| Cow layout | Face to face | 41 | 12 | 21 | 74 |
|  | Tail to tail | 49 | 20 | 10 | 79 |
|  | One row | 75 | 8 | 0 | 83 |
|  | Other | 20 | 1 | 1 | 22 |
|  | Missed values/data not collected | 27 | 8 | 6 | 41 |
| House type | Loose | 162 | 40 | 23 | 225 |
|  | Cubicle | 28 | 7 | 17 | 52 |
|  | Free movement | 17 | 1 | 2 | 20 |
|  | Missed values/data not collected | 5 | 1 | 1 | 7 |
| Farm hygiene^1^ | Unhygienic | 68 | 9 | 3 | 80 |
|  | Satisfactory | 127 | 30 | 23 | 180 |
|  | Neat | 7 | 9 | 11 | 27 |
|  | Missed values/data not collected | 10 | 1 | 1 | 12 |
| Feeding and/grazing for adult animals | Zero grazing (roughage with supplement) | 168 | 35 | 24 | 227 |
|  | Partial grazing (roughage with limited supplement) | 38 | 12 | 11 | 61 |
|  | Grazing only (no supplement) | 1 | 0 | 0 | 1 |
|  | Missed values/data not collected | 5 | 2 | 3 | 10 |
| Breeding strategy | Artificial Insemination | 148 | 32 | 18 | 198 |
|  | Own bull | 33 | 11 | 17 | 61 |
|  | Borrowed bull | 26 | 2 | 0 | 28 |
|  | Missed values/data not collected | 5 | 4 | 3 | 12 |
| Feeding trough | Separate for each animal | 160 | 30 | 30 | 220 |
|  | Common - one trough for all | 20 | 13 | 2 | 35 |
|  | Common - one trough for two or more animals | 24 | 2 | 3 | 29 |
|  | No feeding trough at all e.g. the floor is used | 3 | 0 | 0 | 3 |
|  | Missed values/data not collected | 5 | 4 | 3 | 12 |
| Watering trough | Separate for each animal | 170 | 27 | 25 | 222 |
|  | Common - one trough for all | 28 | 15 | 9 | 52 |
|  | Others | 9 | 3 | 1 | 13 |
|  | Missed values/data not collected | 5 | 4 | 3 | 12 |
| Regular deworming | Yes | 125 | 38 | 30 | 193 |
|  | No | 81 | 10 | 5 | 96 |
|  | Missed values/data not collected | 6 | 1 | 3 | 10 |
| Regular vaccination | Yes | 166 | 40 | 33 | 239 |
|  | No | 39 | 6 | 3 | 48 |
|  | Missed values/data not collected | 7 | 3 | 2 | 12 |

^1^Unhygienic: soiled floor with still waste drainage, bad odour and unethical to see

Satisfactory: waste drained well, tolerable odour

Neat: waste drained well, disposed far away and thus has no bad odour, floor is clean
