# Supplementary material for: The variable prevalence of bovine tuberculosis among dairy herds in Central Ethiopia provides opportunities for targeted intervention
Source: PLoS One. 2021 Jul 2;16(7):e0254091. doi: 10.1371/journal.pone.0254091 (PMC8253440; doi:10.1371/journal.pone.0254091)
Supplement: S2 Table — (DOC) [file pone.0254091.s003.doc]

| S2 Table. Description of risk factors |  |  |
| --- | --- | --- |
| Risk factors | Level | Description |
| Herd size | (4,20] | Exclusive of 4 but inclusive of 20 |
|  | (20,37] |  |
|  | (37,168] |  |
| Age (yrs) | (0.1,2] | Exclusive of 0.1 but inclusive of 2 |
|  | (2,4] |  |
|  | (4,6] |  |
|  | (6, 8] |  |
|  | (8,10] |  |
| Source | Purchased | Bought from market or farm |
|  | On farm bred | Reared at the farm |
| Breed | Cross and exotic | Cross : Pure Holstein Friesian X Zebu, Jersey X Zebu , Exotic:pure HF& Jersey |
|  | Zebu | Indigenous local Zebu breed |
| Sex | Female |  |
|  | Male |  |
| Farm age (yrs) | (4,20] | Farm age since establishment |
|  | (20,35] |  |
|  | (35,68] |  |
| bTB history at farm | Yes | Yes: bovine TB detected during tuberculin testing the last three years |
|  | No | No: Not detected |
| Contact with other domestic animals | Yes | **Presence of possible contact between herd and other domestic animals (e.g. goat/swine/cat/sheep/swine/equine/poultry)** |
|  | No | **No possible contact between herd and other domestic animals (e.g. goat/swine/cat/sheep/swine/equine/poultry)** |
| Stocking density (no. cattle/m^2^) | High | High: > 0.5m^2^ |
|  | Satisfactory | Satisfactory: equals ~0.5m^2^ : one cattle need (1m *2m ) 2m^2^ area (1/2=0.5m^2^) |
|  | Less | Less: < 0.5m^2^ |
| Ventilation | Very good | Above half of the four sides of the wall opened/meshed |
|  | Satisfactory | Above half of the two sides of the wall opened/meshed |
|  | Poor | Closed wall with few windows |
| Viral disease outbreak | Yes | Experience of the incidence of viral disease (FMD, LSD, BVD, etc) in cattle during the last one calendar year |
|  | No | No experience of the incidence of viral disease (FMD, LSD, BVD, etc) in cattle during the last one calendar year |
| Biosecurity measures | Absent | No measures (e.g. visitors allowed) taken to minimize possibility of disease transmission |
|  | Present | Measures (e.g. no visitors allowed) taken to minimize possibility of disease transmission |
| Neighbor herd | Yes | Adjacent herd present |
|  | No | No adjacent herd |
| House type | Free movement | Cows are not restrained and are able to move freely within barn, to enter and leave |
|  | Loose | **Cows are confined together on a platform and secured at neck by rope or neck chains** |
|  | Cubicle | Each cow has a separate stall with or without neck tie |
| Regular de-worming | Yes | Practice of de-worming of the herd once/twice in a year |
|  | No | No practice of de-worming of the herd once/twice in a year |
| Stages of lactation (months) | **(0,2]** |  |
|  | (2,4] |  |
|  | (4,8] |  |
