# Supplementary material for: The variable prevalence of bovine tuberculosis among dairy herds in Central Ethiopia provides opportunities for targeted intervention
Source: PLoS One. 2021 Jul 2;16(7):e0254091. doi: 10.1371/journal.pone.0254091 (PMC8253440; doi:10.1371/journal.pone.0254091)
Supplement: S3 Table — (DOC) [file pone.0254091.s004.doc]

**S3 Table. Collinearity test of candidate explanatory variables**

Variance Inflation Factor (VIF)

GVIF Df GVIF^(1/(2*Df))

| herd_size 3.461112 2 1.363967  age 1.262056 4 1.029520  sex 1.128874 1 1.062485  breed 1.336601 1 1.156115  source 1.141937 1 1.068614  history 1.643870 1 1.282135  fage^a^ 2.815937 2 1.295405  density 1.913065 2 1.176068  bsecurity 1.676919 1 1.294959  neigbour 1.205207 1 1.097819  htype^b^ 2.390506 2 1.243433  ventilation 2.629124 2 1.273365 |
| --- |
|  |

^a^ Farm age;^b^ house type
