# Supplementary material for: The variable prevalence of bovine tuberculosis among dairy herds in Central Ethiopia provides opportunities for targeted intervention
Source: PLoS One. 2021 Jul 2;16(7):e0254091. doi: 10.1371/journal.pone.0254091 (PMC8253440; doi:10.1371/journal.pone.0254091)
Supplement: S4 Table — (DOC) [file pone.0254091.s005.doc]

**S4 Table. Summary of the global and candidate GLMM models (Candidate models in bold)**

| Model | Herd_size | age | breed | sex | fage^a^ | source | history | biosecurity | htype^b^ | ventilation | neighbour | density | htype*ventilation | history*source | df | AIC | Δ AIC | w |
| --- | --- | --- | --- | --- | --- | --- | --- | --- | --- | --- | --- | --- | --- | --- | --- | --- | --- | --- |
| **Global** | **+^s^** | **+^s^** | **+^s^** | **+** | **+** | **+** | **+^s^** | **+** | **+** | **+** | **+** | **+** | **+** | **+** | **30** | **1727.5** | **0** | **0.262855749** |
| Model 1 | +^s^ | +^s^ | +^s^ | + | + | + | +^s^ | + | + | + ^s^ | + | + | - | - | 23 | 1730.507 | 3.007 | 0.058446126 |
| Model 2 | + | + | + | + | _ | + | + | + | + | + | + | - | + | - | 23 | 1751.2 | 23.7 | 1.87641E-06 |
| Model 3 | + | + | + | _ | _ | + | + | + | + | + | + | - | + | - | 22 | 1754.045 | 26.545 | 4.52422E-07 |
| Model 4 | + ^s^ | + ^s^ | + ^s^ | _ | _ | + | + ^s^ | + | + | + | - | - | + | - | 21 | 1818.981 | 91.481 | 3.58815E-21 |
| Model 5 | + ^s^ | + ^s^ | + ^s^ | _ | _ | + | + ^s^ | _ | + | + | - | - | + | - | 20 | 1818.191 | 90.691 | 5.32619E-21 |
| **Model 6** | **+ ^s^** | **+ ^s^** | **+ ^s^** | **_** | **+** | **+** | **+ ^s^** | **_** | **+** | **+** | **-** | **-** | **+** | **-** | **22** | **1728.52** | **1.02** | **0.157843715** |
| Model 7 | + ^s^ | + ^s^ | + ^s^ | _ | _ | + | + ^s^ | + | + | + ^s^ | _ | + ^s^ | - | - | 19 | 1796.757 | 69.257 | 2.40298E-16 |
| **Model 8** | **+** | **+** | **+** | **+** | **+** | **+** | **+** | **+** | **+** | **+** | **+** | **_** | **_** | **_** | **29** | **1730** | **2.5** | **0.075309433** |
| **Model 9** | **+** | **+** | **+** | **+** | **+** | **+** | **+** | **+** | **+** | **+** | **+** | **_** | **+** | **+** | **28** | **1730** | **2.5** | **0.075309433** |
| Model 10 | + | + | + | _ |  | + | + | _ | + | + | + | _ | + | + | 26 | 1749.622 | 22.122 | 4.13034E-06 |
| Model 11 | + | + | + | _ | _ | + | + | _ | _ | + | + | + | _ | _ | 17 | 1752.327 | 24.827 | 1.06808E-06 |
| Model 12 | + | + | + | _ | _ | + | + | _ | _ | + | + | _ | _ | _ | 15 | 1731.347 | 3.847 | 0.038401841 |
| Model 13 | + | + | + | + | + | + | + | + | + | + | + | + | _ | + | 24 | 1733.8 | 6.3 | 0.011263928 |
| **Model 14** | **+** | **+** | **+** | **+** | **+** | **+** | **+** | **+** | **+** | **+** | **+** | **+** | **_** | **+** | **22** | **1729** | **1.5** | **0.124164264** |
| **Model 15** | **+** | **+** | **+** | **+** | **+** | **+** | **+** | **_** | **+** | **+** | **+** | **+** | **_** | **+** | **26** | **1729.7** | **2.2** | **0.087497078** |
| Model 16 | + | + | + | + | + | + | + | + | + | _ | + | + | _ | + | 23 | 1804.6 | 77.1 | 4.76064E-18 |
| Model 17 | + | + | + | + | + | + | + | + | + | + | _ | + | _ | + | 22 | 1731.7 | 4.2 | 0.032188376 |
| Model 18 | + | + | + | + | + | + | + | + | + | + | + | + | _ | + | 24 | 1731.3 | 3.8 | 0.039314971 |
| Model 19 | + | + | + | _ | _ | + | + | _ | _ | _ | _ | + | _ | + | 24 | 1793.8 | 66.3 | 1.09499E-15 |
| Model 20 | + | + | + | _ | _ | _ | + | _ | _ | + | _ |  | _ | _ | 13 | 1816 | 88.5 | 1.59289E-20 |

^S^ significant; ^a^ farm age; ^b^ house type
