# Supplementary material for: The variable prevalence of bovine tuberculosis among dairy herds in Central Ethiopia provides opportunities for targeted intervention
Source: PLoS One. 2021 Jul 2;16(7):e0254091. doi: 10.1371/journal.pone.0254091 (PMC8253440; doi:10.1371/journal.pone.0254091)
Supplement: S1 Questionnaire — (DOC) [file pone.0254091.s006.doc]

**MINISTRY OF LIVESTOCK AND FISHERIES**

**NATIONAL ANIMAL HEALTH DIAGNOSTIC AND INVESTIGATION CENTER**

**Questionnaire for collection of epidemiological data of bovine tuberculosis in central Ethiopia,**

**under the ETHICOBOTS project**

PART 0: Identification

1. Questionnaire number____ 2. Region____________ 3. District________ 4. Kebele_____________

5. Farm ID (ETHICOBOTS) ________

Date of the interview (DD/MM/YYYY; Ethiopian calendar) ____/____/________

**Interview performed by Enumerator: ______________________________**

**Supervisor name: ______________________________**

Information provided is confidential and will not be disclosed/ transferred to other party without the consent of the respondents. Answering the questions depends only on your good. Please answer the questions as accurate as possible. Thank you for your collaboration in advance.

1. **GENERAL INFORMATION**
2. Farm location (GPS, coordinates in UTM): way point-----X(E)__________Y(N)___________Alt ____
3. Dairy farm name: _______________________
4. Name of respondent___________________, Position _____________ 1) manager, 2) owner, 3) Other
5. Owner’s name/manager: _________________Phone (fixed) _________ (Mobile) _________ E-mail:________
6. Respondent’s age:_____________ Sex: 1) Male 2) Female
7. Educational level of the respondent? _______________years of schooling, 0=illiterate, 99=religious
8. Respondent’s religion 1) Orthodox 2) Protestant 3) Muslim 4) Other
9. Ownership of the farm; 1) Private 2) Government 3) cooperative 4) share
10. Number of farm workers living on site Male:_________ Female:_______________
11. Demographic characteristics of the farm household (of the respondent)

| No | **Name of household member (start with respondent)** | | **Age** | **Sex**  **Code A** | **Education**  **Code B** | **Participation in dairy farm (1=Yes, 0=no)** |
| --- | --- | --- | --- | --- | --- | --- |
| 1 |  | |  |  |  |  |
| 2 |  | |  |  |  |  |
| 3 |  | |  |  |  |  |
| 4 |  | |  |  |  |  |
| 5 |  | |  |  |  |  |
| 6 |  | |  |  |  |  |
| **Code A**  0. Female  1. Male | | **Code B:** 0. None/Illiterate  1. Adult education or 1 year of education  * Give other education in years 99. Religious education | | | | |

11. Total number of farm workers? hired___________; family ____________Total_____________

12 Demographic characteristics of farm workers (hired and family members) (Note: for institution large farmers such as universities, research, keep lists separately in paper having the following variables)

| No | **Name of farm worker** | | **Employment status**  Code A | | **Age** | **Sex**  **Code B** | **Education**  **Code C** | | **Position**  **Code D** | **Earning per month in Birr** | | | **months of service on the farm** |
| --- | --- | --- | --- | --- | --- | --- | --- | --- | --- | --- | --- | --- | --- |
| 1 |  | |  | |  |  |  | |  |  | | |  |
| 2 |  | |  | |  |  |  | |  |  | | |  |
| 3 |  | |  | |  |  |  | |  |  | | |  |
| 4 |  | |  | |  |  |  | |  |  | | |  |
| 5 |  | |  | |  |  |  | |  |  | | |  |
| 6 |  | |  | |  |  |  | |  |  | | |  |
| 7 |  | |  | |  |  |  | |  |  | | |  |
| 8 |  | |  | |  |  |  | |  |  | | |  |
| 9 |  | |  | |  |  |  | |  |  | | |  |
| 10 |  | |  | |  |  |  | |  |  | | |  |
| **Code A**   1. Hired   0. Family member | | **Code B**  1. Male  0. Female | | **Code C**  0. None/Illiterate  1. Adult education or 1 year of education  * Give other education in years  99. Religious education | | | | **Code D**  1. Manager  2. Barn worker  2. Store man  3. Guard | | |  | 4. Herd keeper  5. Milker  6. Secretary  7. Other, specify | |

13. Herd structure:

| **Herd composition** | **Pure HF** | **HF Z cross** | **Jersey** | **J Z cross** | **Local** | **Total** |
| --- | --- | --- | --- | --- | --- | --- |
| Calf (0-1yr) |  |  |  |  |  |  |
| Heifers |  |  |  |  |  |  |
| Bullock/Steers (1-2 yrs) |  |  |  |  |  |  |
| Cows |  |  |  |  |  |  |
| Bull/Oxen |  |  |  |  |  |  |
| Total |  |  |  |  |  |  |

*HF-Holstein Frisian, Z- Zebus, JZ - Jersey Zebu cross,

14 Species other than cattle in the farm:

| Species | Number | Possible contact with the dairy cattle | |
| --- | --- | --- | --- |
|  |  | Yes | No |
| Sheep |  |  |  |
| Goats |  |  |  |
| Equine* |  |  |  |
| Dogs |  |  |  |
| Cat |  |  |  |
| Swine |  |  |  |
| Poultry |  |  |  |
| Total |  |  |  |
| None |  |  |  |

*Mention the species: horse, mule or donkey in the remark column

1. **FARM ANTECEDENTS**

15. When did the farm start? (Month, year in E.C) --/----

16. How started this dairy farm its business?

1) Owner bought existing farm/enterprise; 2) Owner established the farm by her/himself; 3) Owner inherited the farm; 4) other (specify) __________

17. How does the farm get replacement stock (multiple options possible)?

1) AI, 2) Use of own bull, 3) By purchasing, 4) Use of bull from other farm, 5) Gift, 6) Government heifer/bull programme 7) Other (specify) _________

18. What was the cattle herd size two years ago? ____________

19. Have you introduced cattle to your farm in the last two calendar years? A) Yes B) No

If ‘yes’, how? A) Purchase B) gift C) calves (new born) D) other

1. a) Complete the following animal details if your answer to question number 19 is choice A or B

| Animal source | | | | | | | | Purpose (trading,  Rearing, etc) | Date animal  entered farm (MM/YY) | Contact address |
| --- | --- | --- | --- | --- | --- | --- | --- | --- | --- | --- |
| Region | City/  town | sub city | District | Kebele | Farm name | Ethicobots  animal ID | ID from  the source |
|  |  |  |  |  |  |  |  |  |  |  |
|  |  |  |  |  |  |  |  |  |  |  |
|  |  |  |  |  |  |  |  |  |  |  |
|  |  |  |  |  |  |  |  |  |  |  |
|  |  |  |  |  |  |  |  |  |  |  |
|  |  |  |  |  |  |  |  |  |  |  |
|  |  |  |  |  |  |  |  |  |  |  |
|  |  |  |  |  |  |  |  |  |  |  |
|  |  |  |  |  |  |  |  |  |  |  |
|  |  |  |  |  |  |  |  |  |  |  |
|  |  |  |  |  |  |  |  |  |  |  |

1. Complete the table below if you have sold or given cattle to other farm (s) or someone.

| Destination | | | | | | Animal ID when sold/given | Purpose | Date animal sold/given (MM/YY) | Contact address |
| --- | --- | --- | --- | --- | --- | --- | --- | --- | --- |
| Region | City/ town | District | Sub city | Kebele | Farm name |
|  |  |  |  |  |  |  |  |  |  |
|  |  |  |  |  |  |  |  |  |  |
|  |  |  |  |  |  |  |  |  |  |
|  |  |  |  |  |  |  |  |  |  |
|  |  |  |  |  |  |  |  |  |  |
|  |  |  |  |  |  |  |  |  |  |
|  |  |  |  |  |  |  |  |  |  |
|  |  |  |  |  |  |  |  |  |  |
|  |  |  |  |  |  |  |  |  |  |
|  |  |  |  |  |  |  |  |  |  |
|  |  |  |  |  |  |  |  |  |  |

1. **FARM MANAGEMENT /HUSBANDRY**

**21. Housing condition**

- 1. What type of house is in use (answered by the interviewer)? A) loose house allowing free movement, B) loose house with tie, C) housing with tie in cubicle, C) housing without tie in cubicle, D) housing shared with people, E) other (specify)________
  2. What is the housing situation of calves? A) separate pen, B) share same barn but no close contact (at least 3m apart) with other animals, C) mixed with others
  3. Do sick animals have isolation pen? A) Yes, B) No
  4. How is the cow layout/arrangement in the house, A) face to face, B) tail to tail, C) one row, D) other (specify)____________
  5. Size of the house/barn?
     1. Floor surface area (LxW)? _____________________ (m^2^).
     2. Indoor height (floor to average point of ceiling)? _________________meter.
  6. Wall condition? A) Solid, B) solid with few windows, C) half open, D) full open
  7. How often do you dispose manure from the barn? A) daily, B) every other day, C) weekly, D) other (specify)___________
  8. What method you use for manure disposal? A) Septic tank, B) Accumulated in open pit, C) Accumulated in the dairy compound, D) used as organic fertilizer/fuel (In Amharic: *kubet*)
  9. How do you (interviewer) rate the ventilation situation? A) Poor (closed wall with few windows), B) Satisfactory (above half of the two sides of the wall opened/meshed), C) very good (above half of the four sides of the wall opened/meshed, and through the roof)
  10. How do you (interviewer) rate the light exposure situation? A) Poor (light enters only through few windows, B) Satisfactory (morning and afternoon light enters through opened opposite side walls, C) very good (morning and evening light enters through any of the four side, through the roof)
  11. How do you (interviewer) rate the overall waste drainage system? A) Poor (floor is levelled prohibiting one side flow or waste flows, and not disposed far away), B) Satisfactory (slant floor, waste easily drains, waste disposed far away), C) Very good (slant floor, waste easily drains, wastes disposed into disposal pit or septic tank, tolerable odour)
  12. How do you (interviewer) rate the overall hygiene of the farm? A) Unhygienic (soiled floor with still waste drainage, bad odour and unethical to see) B) Satisfactory (waste drained well, tolerable odour), C) Neat (waste drained well, disposed far away and thus has no bad odour, floor is clean)

**22. Feed and Water**

1. Do you feed colostrum to neonates? 1) Yes 0) No

If ‘yes’,

i) What is the time of first feeding after birth? 1) Before 6 hrs, 2) 6-12 hrs, 3) 12-24 hrs, 4) after 24 hrs

ii) What is the duration of feeding? 1) For 24 hrs, 2) for 24 hrs to 4 days, 3) for greater than 4 days

1. How do you feed calf with milk? 1) bucket/bottle feeding from bulk milk, 2) bucket/bottle feeding from dam milk, 3) suckling
2. How is the feeding condition for adult animals? 1) Zero grazing (roughage with supplement only), 2) partial grazing (roughage with limited supplement), 3) Grazing only (no supplement)
3. Feeding schedule for roughage, 1) all time, 2) three times a day 3) two times a day 4) once a day 5) Other (specify) ______
4. What type of feed do you feed to your cattle in addition to the roughage (as a supplement)? A) Brewery by product, 1) Yes 0) No, B) wheat bran, 1) Yes 0) No, C) molasses, 1) Yes 0) No, D) formulated ration from feed plant, 1) Yes 0) No, E) homemade 1) Yes 0) No, F) Others (specify)_______ 1) Yes 0) No, G) Cake 1) Yes 0) No
5. Feeding schedule for supplement, 1) three times a day, 2) two times a day 3) once a day 4) every other day, 5) Other (specify)______
6. Amount of feed purchased during the last 12 months?

| Type of feed | Quantity purchased  ( last 12 months ) | Unit | Unit price |
| --- | --- | --- | --- |
| Molasses |  |  |  |
| Bran |  |  |  |
| Cake |  |  |  |
| Hay |  |  |  |
| Crop residue |  |  |  |
| Brewery by product |  |  |  |
| Mineral lick |  |  |  |
| Salt |  |  |  |

1. How do you use a feeding trough? 1) separate for each animal, 2) common-one trough for all, 3) no feeding trough at all e.g. the floor is used 4) common-one trough for two or more animals
2. Do you produce improved forage? 1) Yes 0) No

If ‘Yes’, please fill the following information on forage production on this farm:

| Type of feed | Area (sq.m) | Estimate price if it would have been purchased |
| --- | --- | --- |
| Alfalfa |  |  |
| Elephant grass |  |  |
| Trulecern tree |  |  |
| Napier grass |  |  |
| Other, specify |  |  |

j) How do you use a watering trough?

1) Separate for each animal, 2) common-one trough for all, 3) others (specify) _____________

k) What is your water source (s)? 1) Tap water, 2) well water, 3) Stream, 4) river, E) other (specify)______

l) How frequently are the animals given access to water? 1) All time, 2) four times a day, 3) three times a day, 4) twice a day, 5) Once a day, 6) as necessary, 7) other specify __________________

m) What is your monthly expense for water supplied to the cattle on farm? _______________ Birr

n) What is your monthly expense for electricity bill related to dairy business (for office, barn,…etc) _____Birr

o) What is the cost of fuel and oil related to dairy business during the last twelve months? __________ Birr

1. **FARM BIO-SECURITY**

23. How is the farm enclosure (response by interviewer)? 1) Complete enclosure, 2) partial enclosure, 3) not fenced at all (free movement (in and out) and possibility for cattle to mix with other cattle herds)

24. Which service does the farm mainly use while the cows get heat? 1) AI, 2) own bull, 3) borrowed bull

25. If you borrow bulls, how often do you get the service? 1) Every time any of your cows get heat, 2) only when AI is not possible, C) Rarely

26. Do you know the bovine TB status of the herd you borrow the bull from? 1) Yes 0) No

27. Do you share vets, AI technicians or attendants with neighbouring farms? 1) Yes, 0) No

28. Do wildlife have access to the farm/herd (hyrax, genet, warthog, antelope etc)? 1) Yes 0) No

If yes, mention species of wildlife: _______________________

29. Do dogs and/or cats enter the farm? 1) Yes 0) No

30. How is the interaction of animals with neighbouring herds? 1) share pasture 2) share water 3) share both pasture and water, 4) possibility of direct contact through boundaries although there is no sharing of water and pasture, 5) indirect contact through sewage, manure, fomites, visitors etc., 6) no possibility of contact at all

31. Does any of the employees on farm have his/her own farm (cattle/poultry)? 1) Yes 0) No 2) I don't know

32. Does the farm have latrines/sanitation facility for the farm workers? 1) Yes 0) No

33. Has any animal in the herd had chronic cough/chronic body wastage during the last six months? 1) Yes 0) No

34. Has this farm been tested for bovine TB during the last three years? 1) Yes, 0) No

35. If ‘yes’,

1. When was the test done (E.C)? _____________
2. Was there any positive animals identified by the test? 1) Yes 0) No
3. If ‘yes’, what happened afterwards to those bovine TB positive animals? 1) slaughtered, 2) sold, 3) segregated, 4) No action (animal(s) remained in the herd)

36. Do you take any measures to minimize possibility of disease transmission? A) Yes B) NO

If ‘yes’, which measures? (Multiple options possible)

A) No visitors allowed,

B) Only AI technicians, vets or special guests are allowed

C) Visitor is subjected to use disinfectants at the entry and exit,

D) No access for wildlife, cats and/or dogs to the farm and feed storage,

E) The farm and feed storage has no access for wildlife but for dogs and/or cats,

F) No knowledge on bio-security and thus no measure taken at all,

G) Restrictions on herd grazing/mixing with other herds

H) Practice of burying/ burning fetal membrane and dead bodies

**E. IMMUNOSUPPRESSANT FACTORS**

1. Do you practice regular de-worming of the herd? A) Yes, B) No

If yes, how frequent? A) Twice a year B) Once a year C) others (Specify) __________

1. How often your animals get health care /service? A) regularly, B ) only when ill health situation exist in the herd, others (specify)____________________
2. Do you vaccinate your cattle? A) Yes, B) No

If yes, i) How? A) Regularly, B) when there is an outbreak

ii) For which diseases? A) FMD, B) LSD, C) Anthrax, D) Blackleg, E) Pasteurellosis, F) Other (specify)____

1. How often did you experience the incidence of viral disease (FMD, LSD, BVD, etc) in the cattle during the last one calendar year? A) Just once B) Twice C) not at all
2. In your opinion (interviewer), rate the following stressors based on a 5-point scale where l is zero contribution and 5 high contribution (consider points in brackets and circle one number).

|  | Housing (based on light, ventilation, floor surface condition) | 1 | 2 | 3 | 4 | 5 |
| --- | --- | --- | --- | --- | --- | --- |
|  | Stocking density (based on the standard density) | 1 | 2 | 3 | 4 | 5 |
|  | Diseases (previous exposure for viral diseases, other diseases/syndromes) | 1 | 2 | 3 | 4 | 5 |
|  | Feeding (based on the available feed such as roughage, supplement, grazing) | 1 | 2 | 3 | 4 | 5 |
|  | Watering (based on availability) | 1 | 2 | 3 | 4 | 5 |

Thank you for your time and Cooperation
