# Supplementary material for: The variable prevalence of bovine tuberculosis among dairy herds in Central Ethiopia provides opportunities for targeted intervention
Source: PLoS One. 2021 Jul 2;16(7):e0254091. doi: 10.1371/journal.pone.0254091 (PMC8253440; doi:10.1371/journal.pone.0254091)
Supplement: Attachment — Submitted filename: PONE-D-20-28942_reviewer-1.pdf [file pone.0254091.s012.pdf]

PLOS ONE

High level of bovine tuberculosis in dairy herds of central Ethiopia: a call for intervention

--Manuscript Draft--

**Manuscript Number:**	PONE-D-20-28942
**Article Type:**	Research Article
**Full Title:**	High level of bovine tuberculosis in dairy herds of central Ethiopia: a call for intervention
**Short Title:**	High level of bovine tuberculosis in dairy herds of central Ethiopia
**Corresponding Author:**	Gizat Almaw<br>National Animal Health Diagnostic and Investigation Center, Ethiopia<br>Sebeta, ETHIOPIA
**Keywords:**	Bovine tuberculosis, Dairy cattle, Prevalence, Risk factors, Ethiopia
**Abstract:**	Bovine tuberculosis (bTB) is an important disease for dairy productivity, as well as having the potential for zoonotic transmission. Previous studies on bTB in the dairy sector in central Ethiopia have been fragmented and limited in coverage. Here we carried out a cross sectional one-stage cluster sampling survey to estimate the prevalence of bTB in dairy farms in six areas of central Ethiopia. The survey, which was by far the largest in the area up to date, took place from March 2016 to May 2017 and included Tuberculin testing and collection of additional herd and animal level data by questionnaire to identify potential risk factors contributing to bTB transmission. We applied the Single Intradermal Cervical Comparative Tuberculin (SICCT) test using >4mm cut-off for considering an individual animal as positive for bTB; at least one reactor animal was required for a herd to be considered bTB positive. Two hundred ninety-nine dairy herds in the six study areas were randomly selected, from which 5,675 cattle were tested. The overall prevalence of bTB after standardisation for herd-size in the population was 54.4% (95% CI 48.7-60%)at the herd level, and it was 24.5% (95% CI 23.3-25.8) at the individual animal level. A Generalized Linear Mixed Model (GLMM) with herd and area as random effect was used to explore risk factors associated with reaction status. We found that herd size, age, bTB history at farm, and breed were significant risk factors for animals to be SICCT positive. Animals from large herds had ten times the odds of bTB detection (OR:10, p-value:<0.001) as compared to animals from small herds. The effect of age was strongest for animals 8-10 years of age (the oldest category) having 9.2 times the odds of being reactors(OR:9.2,p-value:<0.001)compared to the youngest category. The other identified significant risk factors were bTB history at farm (OR:3.2, p-value:0.02) and cattle breed (OR:3, p-value: 0.01). Our study demonstrates a high prevalence of bTB in central Ethiopia but with a large variation in within-herd prevalence between herds, findings that lays an important foundation for the future development of control strategies.
**Order of Authors:**	Gizat Almaw
	Andrew JK Conlan
	Gobena Ameni
	Balako Gumi
	Alemseged Alemu
	Sintayehu Guta
	Solomon Gebre
	Abebe Olani
	Abebe Garoma
	Dereje Shegu
	Letebrhan Yimesgen

Powered by Editorial Manager® and ProduXion Manager® from Aries Systems Corporation

	Demeke Nigussie
	James LN Wood
	Tamrat Abebe
	Adane Mihret
	Stefan Berg
**Additional Information:**	
**Question**	**Response**
**Financial Disclosure**<br><br>Enter a financial disclosure statement that describes the sources of funding for the work included in this submission. Review the submission guidelines for detailed requirements. View published research articles from *PLOS ONE* for specific examples.<br><br>This statement is required for submission and **will appear in the published article** if the submission is accepted. Please make sure it is accurate.<br><br>**Unfunded studies**<br>Enter: *The author(s) received no specific funding for this work.*<br><br>**Funded studies**<br>Enter a statement with the following details:<br>• Initials of the authors who received each award<br>• Grant numbers awarded to each author<br>• The full name of each funder<br>• URL of each funder website<br>• Did the sponsors or funders play any role in the study design, data collection and analysis, decision to publish, or preparation of the manuscript?<br>• **NO** - Include this sentence at the end of your statement: *The funders had no role in study design, data collection and analysis, decision to publish, or preparation of the manuscript.*<br>• **YES** - Specify the role(s) played.<br><br>* typeset	The funders had no role in study design, data collection and analysis, decision to publish, or preparation of the manuscript.
**Competing Interests**<br><br>Use the instructions below to enter a competing interest statement for this submission. On behalf of all authors,	The authors have declared that no competing interests exist.

disclose any competing interests that could be perceived to bias this work—acknowledging all financial support and any other relevant financial or non-financial competing interests.<br><br>This statement **will appear in the published article** if the submission is accepted. Please make sure it is accurate. View published research articles from *PLOS ONE* for specific examples.<br><br>**NO authors have competing interests**<br><br>Enter: *The authors have declared that no competing interests exist.*<br><br>**Authors with competing interests**<br><br>Enter competing interest details beginning with this statement:<br><br>*I have read the journal's policy and the authors of this manuscript have the following competing interests: [insert competing interests here]*<br><br>* typeset	
**Ethics Statement**<br><br>Enter an ethics statement for this submission. This statement is required if the study involved:<br><br>• Human participants<br>• Human specimens or tissue<br>• Vertebrate animals or cephalopods<br>• Vertebrate embryos or tissues<br>• Field research<br><br>Write "N/A" if the submission does not require an ethics statement.<br><br>General guidance is provided below. Consult the submission guidelines for detailed instructions. **Make sure that all information entered here is included in the Methods section of the manuscript.**	The project was approved by Institutional Review Board (Project Reg.No PO46/14) and National Research Ethics Review Committee (NRERC No. 3.10/800/07).

**Format for specific study types**<br><br>**Human Subject Research (involving human participants and/or tissue)**<br>• Give the name of the institutional review board or ethics committee that approved the study<br>• Include the approval number and/or a statement indicating approval of this research<br>• Indicate the form of consent obtained (written/oral) or the reason that consent was not obtained (e.g. the data were analyzed anonymously)<br><br>**Animal Research (involving vertebrate animals, embryos or tissues)**<br>• Provide the name of the Institutional Animal Care and Use Committee (IACUC) or other relevant ethics board that reviewed the study protocol, and indicate whether they approved this research or granted a formal waiver of ethical approval<br>• Include an approval number if one was obtained<br>• If the study involved *non-human primates*, add *additional details* about animal welfare and steps taken to ameliorate suffering<br>• If anesthesia, euthanasia, or any kind of animal sacrifice is part of the study, include briefly which substances and/or methods were applied<br><br>**Field Research**<br><br>Include the following details if this study involves the collection of plant, animal, or other materials from a natural setting:<br>• Field permit number<br>• Name of the institution or relevant body that granted permission	
**Data Availability**<br><br>Authors are required to make all data underlying the findings described fully available, without restriction, and from the time of publication. PLOS allows rare exceptions to address legal and ethical concerns. See the PLOS Data Policy and FAQ for detailed information.	Yes - all data are fully available without restriction

A Data Availability Statement describing where the data can be found is required at submission. Your answers to this question constitute the Data Availability Statement and **will be published in the article**, if accepted.<br><br>**Important:** Stating 'data available on request from the author' is not sufficient. If your data are only available upon request, select 'No' for the first question and explain your exceptional situation in the text box.<br><br>Do the authors confirm that all data underlying the findings described in their manuscript are fully available without restriction?	
**Describe where the data may be found in full sentences. If you are copying our sample text, replace any instances of XXX with the appropriate details.**<br><br>• If the data are **held or will be held in a public repository**, include URLs, accession numbers or DOIs. If this information will only be available after acceptance, indicate this by ticking the box below. For example: *All XXX files are available from the XXX database (accession number(s) XXX, XXX.*).<br>• If the data are all contained **within the manuscript and/or Supporting Information files**, enter the following: *All relevant data are within the manuscript and its Supporting Information files.*<br>• If neither of these applies but you are able to provide **details of access elsewhere**, with or without limitations, please do so. For example:<br><br>*Data cannot be shared publicly because of [XXX]. Data are available from the XXX Institutional Data Access / Ethics Committee (contact via XXX) for researchers who meet the criteria for access to confidential data.*<br><br>*The data underlying the results presented in the study are available*	All relevant data are within the manuscript and its Supporting Information files.

from (include the name of the third party and contact information or URL).<br>• This text is appropriate if the data are owned by a third party and authors do not have permission to share the data.<br><br>* typeset	
Additional data availability information:	

Powered by Editorial Manager® and ProduXion Manager® from Aries Systems Corporation

High level of bovine tuberculosis in dairy herds of central Ethiopia: a call for intervention

Gizat Almaw[1,2,*], Andrew JK Conlan[6], Gobena Ameni[4], Balako Gumi[4], Alemseged Alemu[1], Sintayehu Guta[1], Solomon Gebre[1], Abebe Olani[1], Abebe Garoma[1], Dereje Shegu[1], Letebrhan Yimesgen[1], Demeke Nigussie[7], James LN Wood[6], Tamrat Abebe[2], Adane Mihret[2,3], Stefan Berg[5] and the ETHICOBOTS consortium[^]

[1]National Animal Health Diagnostic and Investigation Center, Sebeta, Ethiopia

[2]Department of Microbiology, Immunology and Parasitology, College of Health Sciences, Addis Ababa University, Addis Ababa, Ethiopia

[3]Armauer Hansen Research Institute, Addis Ababa, Ethiopia

[4]Aklilu Lemma Institute of Pathobiology, Addis Ababa University, Addis Ababa, Ethiopia

[5]Bacteriology Department, Animal and Plant Health Agency, Weybridge, United Kingdom

[6]Disease Dynamics Unit, Department of Veterinary Medicine, University of Cambridge, Cambridge, United Kingdom

[7]Ethiopian Institute of Agricultural Research, Addis Ababa, Ethiopia

*Corresponding author

E-mail address: gizatalm@yahoo.com(G Almaw)

^Members of the ETHICOBOTSconsortium are listed in the Acknowledgments

Abstract

Bovine tuberculosis (bTB) is an important disease for dairy productivity, as well as having the potential for zoonotic transmission. Previous studies on bTB in the dairy sector in central Ethiopia have been fragmented and limited in coverage. Here we carried out a cross sectional one-stage cluster sampling survey to estimate the prevalence of bTB in dairy farms in six areas of central Ethiopia. The survey, which was by far the largest in the area up to date, took place from March 2016 to May 2017 and included Tuberculin testing and collection of additional herd and animal level data by questionnaire to identify potential risk factors contributing to bTB transmission. We applied the Single Intradermal Cervical Comparative Tuberculin (SICCT) test using >4mm cut-off for considering an individual animal as positive for bTB; at least one reactor animal was required for a herd to be considered bTB positive. Two hundred ninety-nine dairy herds in the six study areas were randomly selected, from which 5,675 cattle were tested. The overall prevalence of bTB after standardisation for herd-size in the population was 54.4% (95% CI 48.7-60%)at the herd level, and it was 24.5% (95% CI 23.3-25.8) at the individual animal level. A Generalized Linear Mixed Model (GLMM) with herd and area as random effect was used to explore risk factors associated with reaction status. We found that herd size, age, bTB history at farm, and breed were significant risk factors for animals to be SICCT positive. Animals from large herds had ten times the odds of bTB detection (OR:10, p-value:<0.001) as compared to animals from small herds. The effect of age was strongest for animals 8-10 years of age (the oldest category) having 9.2 times the odds of being reactors(OR:9.2,p-value:<0.001)compared to the youngest category. The other identified significant risk factors were bTB history at farm (OR:3.2, p-value:0.02) and cattle breed (OR:3, p-value: 0.01). Our study demonstrates a high prevalence of bTB in central Ethiopia but with a large variation in

within-herd prevalence between herds, findings that lays an important foundation for the future development of control strategies.

Keywords: Bovine tuberculosis, Dairy cattle, Prevalence, Risk factors, Ethiopia

Introduction

Bovine tuberculosis (bTB) is a chronic disease of cattle primarily caused by *Mycobacterium bovis* (*M. bovis*), which has zoonotic potential and can also infect other domestic and wild animals. The disease is prevalent in most of Africa, parts of Asia and the Americas, and in several European countries. Many industrialised countries have managed to reduce or eliminate bTB in their livestock sectors through test-and-slaughter, however significant pockets of infection remain in wildlife [1]. In Africa the disease is endemic largely due to a lack of control measures. This has economic implications for the growth of the livestock sector, especially the dairy sector, and poses the risk of zoonotic TB transmission which is exacerbated by the existence of concomitant infections such as HIV/AIDS [2]. In Ethiopia, the demand for milk is expanding rapidly due to increased urbanization and population pressure; Ethiopia is the second most populous country in Africa with an estimated population of 110 million people[3]. Since the introduction of intensive dairy farming in central Ethiopia in the 1950s to provide the Emperor and his establishment with milk, the dairy sector has steadily increased - especially the last 30 years - trying to meet the demand from increased urbanization and it supplies now with milk and milk products to the city dwellers[4].Although the dairy sector is most developed in

central Ethiopia, urban centers across the country have more recently seen an increase in dairy farming. This most developed dairy belt in Ethiopia is expected to be challenged with diseases of intensification such as bTB [5,6]. This is believed to be associated with mainly two factors: Firstly, a shift from dairy herding with existing local zebu cows to crosses of exotic breeds (mainly Holstein Friesian cows), which produce higher milk yields, have established dairy herds that are likely to be more susceptible to bTB [7, 8]. Secondly, an intensified dairy sector with larger herds has likely increased disease transmission as bTB is thriving in an environment with higher density population. bTB animal prevalence recorded in Ethiopia has ranged from around 3% in smallholder production systems (rearing mainly zebu cattle) up to 48% in intensive dairy productions [5,7, 9,10, 11]and the national average recently estimated to be ~ 5.8% [12]. Tschopp and colleagues [13] estimated the cost of bTB in Ethiopia for the urban livestock production to have ranged from US$500,000–4.9 million between 2005 and 2011. These figures demonstrate a 3.9–6.2% loss per year of the net livestock value (for the year 2005)[13].One target for the Ethiopian government in its 2015-2020 Livestock Master Plan is to transform the dairy sector by increasing the number of crossbred cattle by almost eight times the base-year number [22]. Such expansion comes however with a risk since transmission of infectious diseases, such as bTB, is likely to thrive by intensification [12, 23]. This also raises the concern that bTB may spread to the emerging dairies in the regional towns through trading of high milk yield animals from infected farms in the central regions. As previous studies on bTB in this area had been fragmented and limited in coverage,there was a clear need to carry out a comprehensive survey to understand bTB in dairies in central Ethiopia [12]. Therefore we carried out a large scale survey to assess the current status of bTB prevalence in the established dairy sector in

central Ethiopia and to identify contributing risk factors for the spread of the disease to inform the development of potential control strategies.

Materials and methods

Study areas

Six study areas were selected in the urban areas of central Ethiopia, including Addis Ababa city, and Sebeta, Holeta, Sululta, Sendafa and Bishoftu towns(Fig 1).The selection of these study areas was purposeful. Central Ethiopia, which includes the study areas, was a pioneer for the modern dairy development in Ethiopia with the first number of exotic dairy cattle arriving in the early 1950s as a donation from the United Nations [4]and this area has then over decades established itself as the most developed dairy belt in Ethiopia. The study areas are currently the main milk suppliers for people in Addis Ababa and the surrounding peri-urban areas.

Fig 1. Map of the study areas: Addis Ababa city and Sululta, Sendafa, Holeta, Sebeta, and Bishoftu towns.

Study design

This study was a cross sectional study conducted from March 2016 to May 2017. Lists of herds (the sampling frame) were established at the start of the study in collaboration with district veterinary officers in respective study sites. The term "herd" was used to describe the group of cattle that are housed on a holding at the time of data collection [15].Herds, with the purpose of producing milk and dairy products, having five or more cattle were included and a list of 1,323

herds was established as a sampling frame. The herds were classified as small [5-20], medium [21-37], and large herds [38-168] (168 being the largest herd size in the studied herds)[5].

Inclusion and exclusion criteria for the study herds: Herd size was the criteria used and herds with less than five animals were excluded.

Sample size and sampling

Sample size was determined following one-stage cluster sampling method taking dairy herd as a cluster [16]and every animal in the selected cluster was tested.

$$g = \frac{1.96^2 \{\text{nVC} + \text{Pexp}(1 - \text{Pexp})\}}{nd^2}$$

Where : g = number of herd to be sampled;
n = predicted average number of animals per herd (n = 13);
Pexp = expected prevalence (Pexp = 0.3 from previous study [5]
d = desired absolute precision (d = 0.05);
VC = between - herd variance (VC = 0.233) [34]

When we fitted these numbers to the formula which assumes large population, it gave us 383 farms. It was reduced to 298, because it was adjusted for small population using the formula:

$$g_{adj} = \frac{G * g}{G + g}, [16]$$

Where : G = total number of herds
g = the calculated sample size for large herds

$$g_{adj} = \frac{1323 * 383}{1323 + 383} \approx 298$$

Hence, we tested 299 herds out of 1323 registered herds in the study sites and selection of each herd was random. All animals in the 299 herds (5,675 animals) were tested excluding animals less than 3 months of age and pregnant cattle ≥8 months pregnant.

For herd recruitment and sampling of the 299 herds, proportionate sample was obtained using the formula: (sample size/population size) x stratum size (small, medium or large herd) [16] i.e. 298/1323 = 0.225 x stratum size. In the actual study the fractions for large, medium and small herds were 71% (n=212), 16% (n=49) and 13% (n=38), respectively and those in the overall population were 89%, 7% and 4%. The over-representation of larger farms was due to a greater level of refusal to participate in smaller herds, despite efforts to address this, and numbers were made up in medium and large herds. Direct method of standardisation(adjustment)[16] was employed to adjust for the effect of having a higher representation of larger farms in the crude overall bTB prevalence result.

Single Intradermal Cervical Comparative Tuberculin (SICCT) test

The procedure for SICCT test was adapted from OIE Terrestrial Manual, 2009 (Bovine Tuberculosis) and the supplier of Tuberculin PPD was Prionics, Lelystad, The Netherlands. The injection site used was at the border of the anterior and middle thirds of left side (for consistency) of the neck. Two sites were used, one for bovine PPD (lower site) and the other for avian PPD (upper site). The upper site was 10 cm below the crest and the lower site was 12.5 cm from the upper site, on a line drawn parallel with the line of the shoulder. The selected site of injection was shaved to an adequately sized area for identification of the injection sites and cleansed. Before injection, a fold of skin at each of the intended injection sites and within the clipped area was taken between the forefinger and thumb and measured to the nearest millimeter using the

same digital caliper (0-150mm range) throughout the survey. Then 0.1 ml of Bovine Tuberculin PPD and 0.1ml of Avian Tuberculin PPD was injected intradermally in the lower and upper site, respectively. A correct injection was confirmed by palpating a small pea-like swelling at each injection site. The two injection sites were re-measured after 72 hours by the same person who measured the skin thickness before the injection. For the interpretation, the SICCT was considered positive if the difference was more than 4 mm; inconclusive if between 1 to 4 mm; and negative if the increase in skin thickness at the bovine site of injection was less than 1 mm or equal to the increase in the skin reaction at the avian site of injection.

Farm data collection

Data was collected by trained research assistants through face to face interview with pre-tested structured questionnaire to capture animal and herd-level information. General information including herd structure, farm antecedents, farm management/husbandry, housing/ventilation, animal health (veterinary services) and animal bio-security were recorded. Specific information related to potential risk factors for bTB were recorded including animals age, sex, breed, physiology (pregnancy/stages of lactation/body condition), herd size, cattle sourcing (cattle movements in and out of the herd), bTB history on farm, contacts /interactions with neighboring herd/other domestic animals/wild animals etc. (S1 Questionnaire). GPS data was collected for each herd for mapping bTB prevalence in the study areas (S1 Fig).

Statistical analysis

Data from questionnaires and the tuberculin skin test were curated and coded. All the statistical analysis was performed using RStudio and the R statistical language [17].Based on the Single Intradermal Cervical Comparative Tuberculin (SICCT) test, the animal level and herd level bTB prevalence for Addis Ababa city and surrounding five study areas was described and 95% confidence interval calculated. Using GPS data, bTB prevalence maps were created for the six study areas, each visualizing the bTB burden for large, medium and small herds (S1 Fig).Kruskal–Wallis test was used for comparison of variability in within herd bTB prevalence (%)among studied dairy herds. Sixteen potential risk factors (Table 4) were analyzed and risk was modelled at the individual animal level. Our dataset was hierarchal in nature *i.e.* individual animals were clustered within herds and herds were clustered within study areas. To account for this clustering and deal with variation in prevalence between study areas and in particular between herds, Generalized Linear Mixed Model (GLMM)[32]was used which allowed us to treat herd and study areas as random effects with a binary response as an outcome variable (bTB reactor or not reactor). We used the glmer() function in the lme4 package[18]. The statistical unit of analysis was individual animal. We performed a univariable screen to select variables for inclusion in the multivariable model (Table 4). All variables with a p-value of < 0.20 and those with a high biological relevance were considered as candidate variables for the model building. These candidate explanatory variables were investigated further for collinearity requiring that all selected variables for the multivariable model have a variance inflation factor (VIF) of < 5 [11] (Table 3 in S1 supplementary). Statistical significance was set at the 5% level.

The binomial or, more specifically, the Bernoulli distribution is considered for modeling our binary data[29].To specify the model, we define the binary response variable:

$$Y_i = \begin{cases} 1 & \text{If the animal is positive for bTB,} \\ 0 & \text{Otherwise.} \end{cases}$$

$$Y_i \sim \text{Bin}\ (\text{P}_\text{i})$$

The probability P_i of the ith animal being bTB positive is :

$$\log\left(\frac{\text{P}_\text{i}}{1 - \text{P}}\right) = \beta X_i + \mu_{herd(i)} + \gamma_{area(i)},$$

Where :

β is a parameter of fixed effects,

Xi are explanatory variables values for the ith animal,

$\mu_{herd(i)}$ is the random effect of the herd (which contains animal i),

$\gamma_{area(i)}$ is the random effect of the study area (which contains animal i),

All screened predictors were initially included in the global model, including biologically plausible two-way interactions(Table 4 in S1 supplementary).Breed was considered as potential confounder for herd size. As some confounding is invariably present, and the important issue is how large the confounding effect is, not whether or not it is present [32].We specified a difference of 20 % change in the odds ratioas an indication of confounding[32].The removal of breed from the final model changed the logit of herd size by 9.8% (16.8-15.3)/15.3) for medium herds and by 2% (10.2-10)/10)(data not shown in the table) for large herds, thus no strong confounding was found between the two factors.

The model fit was assessed by the Hosmer-Lemeshow test (binomTools-package) and its classification ability was explored using ROC (receiver-operator curve) analyses (ROCR-package) [19].

Ethical considerations

The project was approved by Institutional Review Board (Project Reg.No PO46/14) and National Research Ethics Review Committee (NRERC No. 3.10/800/07).

Results

Description of the herd demography and characteristics

This study investigated 299 dairy herds (212 small, 49 medium, and 38 large farms) for bTB using the SICCT test in the urban and peri-urban areas of central Ethiopia. In addition, descriptive data on these herds were collected. With regard to ownership of the studied herds, 238(82.9%) herds were owned privately, 31were cooperatives (10.8%), eight were government herds (2.8%) and ten were share companies(3.5%). Twelve herds had no records about ownership. The majority of herds (77.1%) had loose house type and practice zero grazing (roughage with supplement feeding) (78.5%). Artificial insemination was the main breeding strategy for 69% of these farmers, 83% vaccinated their cattle against major diseases, while 67% dewormed their cattle on a regular basis. The herd structure of the studied dairy herds is presented in Table 1 and additional herds characteristics is provided in Table 1 in S1 supplementary.

Table 1. Herd structure of the 299 studied dairy herds

Characteristics	Levels	Herd size			
		Small (n=212)	Medium (n=49)	Large (n=38)	Total (n=299)
Calf (0-1yr)	Crossbreed	381	257	360	998
	Zebu	34	5	1	40
	Exotic (pure)	0	0	1	1
Heifer	Crossbreed	360	191	413	964

	Zebu	15	4	1	20
	Exotic(pure)	0	0	0	0
Cow	Crossbreed	1116	703	1486	3305
	Zebu	37	15	52	104
	Exotic(pure)	8	2	0	10
Bullock/Steers	Crossbreed	17	27	24	68
(1-2 yrs)	Zebu	6	2	3	11
	Exotic (pure)	0	2	1	3
Bull/Oxen	Crossbreed	32	14	31	77
	Zebu	52	11	6	69
	Exotic(pure)	0	0	5	5
**Total cattle**	**Total**	**2058**	**1233**	**2384**	**5675**
Other animals	Sheep	549	310	500	1359
	Goats	99	56	77	232
	Equine	142	24	34	200
	Dogs	260	87	58	405
	Cats	167	69	18	254
	Swine	6	45	1511	1562
	Poultry	5963	6952	7541	20456

Prevalence of bTB in the study population

In total 5,675 cattle from 299 herds were tested by using the SICCT test. Overall there were1,776 reactors (31.3% crude animal prevalence- not adjusted for herd size; 95% CI: 30-33%) in 180 herds (60.9% crude herd prevalence; 95% CI:55.2 -66.2%), with each positive herd having at

least one reactor (Table 2). Sebeta had the highest prevalence (42% at animal level with 95% CI: 38-46% and 74%at herd level with 95% CI: 55-87%) among all six regions whereas Holeta had the lowest prevalence(17% at animal level with 95% CI: 14-20% and 27% at herd level with 95% CI: 13-46%). There was significant variation between study areas in prevalence of tuberculin reactors (χ^2 = 143.18, df = 5, p-value <0.001).

Table 2. Animal and herd level bTB prevalence for 299 dairy herds in the six study areas

**Level**	**Addis Ababa**	**Sebeta**	**Holeta**	**Sululta**	**Sendafa**	**Bishoftu**	**Total**
**Animal level:** % Prev. (95%CI)	32.8(31-35)	42.2(38-46)	16.8 (14 -20)	41.9(38-46)	25.5(22-30)	25.5 (23-28)	31.3(30-33)
Positives	797	250	90	257	134	248	1776
Total number tested	2432	593	537	614	525	974	5675
**Herd level:** % Prev.(95%CI)	63 (55-70)	74 (55-87)	30 (13-46)	60(39-78)	54(33-74)	73.3(50-85)	60.9(54-66)
Positives	100	23	9	15	13	22	182
Total number tested	159	31	30	25	24	30	299

Herd-size specific prevalenceof bTB

The bTB prevalence was stratified on herd size based on the study population (Table 3A). The results showed a different prevalence between herd sizes with a significant increase in prevalence with herd size group. As the recruitment of herds into the study had been somewhat

over-represented of larger herds as compared to the original sampling strategy, it was relevant to standardise the prevalence estimates in the study population. Therefore Table 3B presents herd size specific prevalence of bTB for the standard population (a population we aimed to sample) of all study sites. The overall crude bTB prevalence was higher (31.3%: 95% CI: 30-33) compared with herd size adjusted prevalence (24.5%:95%CI:23.3-25.8) (using direct method of standardisation).The same trend was recorded for the herd level bTB prevalence (Table 3).

Table 3. Prevalence of bTB stratified by herd-size for (A) the study population and (B) the standard population of the study areas

**A**	**Study population**				
	**Herd size group**	**Herds sampled**	**Population**	**bTB positives**	**Prevalence % (95% CI)**
**Animal Level**	Small herds (4,20][a]	212	2058	373	18.1 (16.5-19.4)
	Medium herds (20,37]	49	1233	402	32.6 (30-35.3)
	Large herds (37,168]	38	2384	1001	42.0 (40-43.9)
	Total	299	5675	1776	**31.3 (**30-33)
**Herd Level**	Small herds (4,20]	212	212	108	50.9**(**44.3-57.6)
	Medium herds (20,37]	49	49	41	83.7**(**71-91.5)
	Large herds (37,168]	38	38	33	86.8 **(**72.7-94.2)
	Total	299	299	182	**60.9(**55.2-66.2)

[a]Exclusive of 4 but inclusive of 20

B	Standard population				
	**Herd size group**	**Expected herds sampled**	**Expected population** [a]	**Expected bTB positives** [b]	**Expected Prevalence % (95% CI)**
**Animal Level**	Small herds (4,20]	266	2926	530	18.1 (16.8-19.6)
	Medium herds (20,37]	21	609	199	32.7 (29.1-32.7)
	Large herds (37,168]	11	792	333	42 (38.7-45.5)
	Total	298	4327	1062	**24.5** (23.3.-25.8)
**Herd Level**	Small herds (4,20]	266	266	134	50.4(44.4-56.3)
	Medium herds (20,37]	21	21	18	85.7(65.4-95.0)
	Large herds (37,168]	11	11	10	90.9(62.3-98.4)
	Total	298	298	162	**54.4**(48.7-60)

[a]Expected population = Expected herds sampled * Average population size (for each herd size group)

[b]Expected bTB positives = Expected population * Prevalence in study population (for each herd size group)

Within herd prevalence of bTB

The average within-herd prevalence is heavily skewed by a relatively small proportion of extremely high prevalence herds (illustrated by Figure 2). Within-herd prevalence is multi-modal with the majority of small and medium herds having a prevalence less than the population average. The population mean 31.5% was higher compared to the median 10%, thus indicating a positive skewedness and that a higher proportion of herds (67.9%) had a within herd prevalence less than the population average. Although the average within-herd prevalence does not demonstrate a strong herd-size dependence, there is a marked difference in the distribution with a markedly higher proportion of herds having a prevalence greater than the population average. A greater proportion of large herds (65.8%) (median: 50%) were having within herd prevalence greater than the population average.

Fig. 2. (A) Within-herd bTB prevalence distribution for stratified herds (Visualizing multiple distributions simultaneously) (B) Within-herd bTB prevalence distribution for affected herds (bTB prevalence > 0)

Translating this into numbers: the mean within herd prevalence for all herds was 31.5± 30.7% and a median of 10% (lower quartile0 and 42.5% upper quartile). Stratification on large, medium, and small herds, there was a mean within herd prevalence of 40.6%, 35.1%, and 18.8%, respectively, while the median value for the respective herd size was50%, 33%, and 8.3..In this study, there was a significant difference in within herd prevalence among studied dairy herds (Kruskal–Wallis test: df=2,χ^2=33.295, p value < 0.001).

Risk factor analysis

Sixteen potential risk factors, based on knowledge and understanding of the husbandry system and biological relevance were considered and screened by univariable analysis(Table 4). Twelve variables with p-value of < 0.20 and OR > 1 were selected for multivariable analysis. Contact with other domestic animals, stages of lactation, viral disease outbreak, and regular de-worming did not fulfil the stated criteria and were excluded from analysis. A full description of the measured risk factors is provided in Table 2 in S1 supplementary. Total number of examined animals(3rd column in Table 4) used for analysis of respective risk factor may differ from the overall number of animals tested (N= 5,675) due to missing values.

Table 4. Univariable analysis of potential risk factors for cattle tuberculin reactors

Risk factors	Level	Proportion %(bTB positives/total examined)	OR (95% CI)	P value
Herd size	(4,20]	18.1 (373/2058)	ref	<0.001
	(20,37]	32.6 (402/1233)	2.2 (1.8-2.6)	
	(37,168]	42 (1001/2384)	3.3 (2.8-3.8)	
Age (yrs)	(0.1,2]	21.3 (422/1980)	ref	<0.001
	(2, 4]	33.1 (470/ 1420)	1.8 (1.5-2.1)	
	(4,6]	34.3(376 /1095)	1.9 (1.6-2.3)	
	(6,8]	39.7(224/564)	2.4 (1.9-3)	
	(8,10]	41.6 (82/19)	2.6 (1.9-3.6)	
Source	Purchased	37.5 (344/916)	1.4 (1.2-1.6)	<0.001
	On farm bred	30 (1431/4757)	ref	
Breed	Cross and exotic	32.3 (1757/5431)	5.7 (3.6-9.4)	<0.001
	Zebu	7.8 (19/244)	ref	
Sex	Female	32.4 (1698/5242)	2.2 (1.7-2.8)	<0.001
	Male	18 (78/433)	ref	
Farm age (yrs)	(4,20]	25.4 (695/2736)	ref	<0.001
	(20,35]	36.6 (715/1951)	1.7 (1.4-1.9)	
	(35,68]	30 (213/708)	1.3 (1-1.5)	
bTB history at farm	Yes	40.8 (381/932)	1.4 (1.1-1.6)	<0.001
	No	33.4 (538/1607)	ref	
Contact with other domestic animals	Yes	32.5 (702/2161)	1.04 (0.8-1.2)	0.64
	No	31.5 (254 /806)	ref	
Stocking density(no.	High	39.8 (300/753)	1.2(0.8-1.9)	<0.001

cattle/m^2)	Satisfactory	35.4(34/96)	0.7(0.5-1.1)	
	Less	28.6(1314/4601)	ref	
Ventilation	Very good	28.6 (608/2127)	ref	<0.001
	Satisfactory	29.7 (506/1706)	1(0.9-1.2)	
	Poor	34.9 (548/1572)	1.3(1.2-1.5)	
Viral disease outbreak	Yes	30.6 (851/2784)	ref	0.35
	No	31.2 (867/2728)	0.9(0.8-1.1)	
Biosecurity measures	Absent	32.8 (1349/4109)	1.4 (1.1-1.6)	< 0.001
	Present	26.4 (384/1457)	ref	
Neighbor herd	Yes	31.4 (1527/4857)	1.7 (1.3-2.1)	<0.001
	No	21.5(106/494)	ref	
House type	Free movement	27.2 (94/345)	1.4 (1-1.8)	<0.001
	Loose	34.5 (1329/3856)	1.9 (1.6-2.2)	
	Cubicle	21.4 (281/1313)	ref	
Regular de-worming	Yes	29.2 (1239/4247)	0.8 (0.7-9.9)	<0.001
	No	35.3(428/1212)	ref	
Stages of lactation	(0,2]	34.7 (137/395)	ref	0.28
(months)	(2,4]	36.2(179/494)	1.1(0.8-1.4)	
	(4,8]	39.2(304/776)	1.2(0.6-1.6)	

Multivariable analysis of potential risk factors for positive cattle reactors using GLMM with herd and area as random effect.

Based on their high OR, absence of collinearity and statistical significance (p-value <0.2),twelve variables(Table 4) were considered in the final multivariable model. The final model thus consisted of four variables: herd size, age, bTB history at farm, and breed as significant risk factors for bTB. Animals from large herds had 10times the odds of bTB detection compared to

animals living in small herds. There was also a strong effect of age, with animals 8-10years of age having 9.2times the odds of being reactors compared to the youngest category (Table 5).

Table 5. GLMM multivariable analysis of potential risk factors for bTB positive cattle using herd and area as random effect

Risk factor	Level	OR (95% CI)	P value
Herd size	(4,20]	ref	<0.001
	(20,37]	15.3(5.8-38.7)	
	(37,168]	10 (3.1-32.4)	
Age (yrs)	(0.1, 2]	ref	< 0.001
	(2, 4]	3.1(2.3-4.1)	
	(4,6]	4(2.9-5.5)	
	(6, 8]	5.9 (4.1-8.4)	
	(8,10]	9.2(5.7-15.9)	
bTB history at farm	Yes	3.2 (1.3-7.6)	0.02
	No	ref	
Breed	Crossand exotic	3(1.5-5.8)	0.01
	Zebu	ref	

Discussion

In this study we set out to perform the largest bTB prevalence study so far in dairy farms in central Ethiopia (Fig 1) to get a comprehensive understanding of the scope of the burden of the disease and identify potential risk factors contributing to the transmission of bTB within the study area. This study therefore addressed limitations of previous studies in this area which had been fragmented and limited in coverage. With an overall crude animal prevalence of 31.3% (n=1,776) (herd size adjusted: 24.5%)and a 60.9% (n=180) crude prevalence at herd level (herd size adjusted: 54.4%), we recorded a high level of bTB prevalence. However, there was variation between the six study areas: relatively low prevalence was recorded in Holeta and this could be related to earlier work to control for bTB in selected infected government herds in that area, which at the time were supplying heifers to surrounding farmers [20]. In this survey we also noted significant variation of within-herd bTB prevalence (P-value < 0.05) among the studied dairy herds, whichranged from 0 to 100% and with a mean for all herds of 31.5% ± 30.7. This variability would mean differences in transmission due to husbandry and other risk factors discussed in this paper [25].By herd stratification, large herds recorded the highest within-herd prevalence (mean: 40.6%) and a larger proportion (65.8%) had a within-herd prevalence greater than the population average. Such high herd prevalence could be due to an increased risk of within-herd transmission in farms with larger herd size [33]. This finding is relevant for control measures such as limited test and removal which could be economically viable in the lower prevalence herds.

Risk factors influence transmission and can becategorisedat regional, herd, and animal level [26]and vary across regions for several reasons, such as difference in farm management practices [14]. Analysis of this can be useful to develop a strategy for risk-based surveillance and control for bTB. The present study has identified several risk factors for bTB. Animals from large herds had 10 times the odds of detection compared to those from small herds. Herd size is the most frequently reported risk factor for bTB in Ethiopia and elsewhere [5, 10,11,24,27]. The risk of infection in a herd increases with herd size and this could be due to overcrowding which increases probability of contact between animals in larger herds implying that transmission may be density dependent [25]. High density creates favorable environment for bTB as aerosol is one main route of transmission. The postmortem data collected by Firdessa and colleagues [5] support this as most animals had TB lesions in lungs and/or lung associated lymph nodes. Also, larger herds often have a larger grazing area, which may expose them to greater environmental risk factors (e.g. wildlife reservoir though not confirmed in Ethiopia) and may also expose them to more neighboring herds [27].Although the number of large herds in Ethiopia are few (even in the central part of the country) their impact on bTB transmission is likely to be significant as many of them are highly infected and they are primary suppliers of heifers to smallholder farms as well as of milk to consumers and could therefore be most potential sources of infection. If a future bTB control program in Ethiopia would focus on these farms, such intervention could possibly be financially affordable given their small number and turning them into bTB free herds could potentially have a significant impact on the overall bTB prevalence in the Ethiopian dairy sector.

When looking for other potential risk factors, there was also a strong effect of age. Animals between 6 – 8 years old were having the highest odds of bTB detection (OR: 9.2, 95% CI: 5.7-

15.9) compared to the baseline category, which was the youngest age group. A linear increase between bTB infection and age was reviewed by Broughan *et al*. [25]and observed in slaughterhouse surveillances in cattle in Northern Ireland and Great Britain[28, 29]. The mean age of reactor cattle was 4.4 years (95% CI: 4.29-4.56). Longevity increases probability of exposure and it also increases the chance for development of visible TB lesions and detection in slaughterhouse surveillances. In addition, purchase of older cattle - particularly from high risk areas - could increase the risk of introducing bTB in a herd. Instead, the adoption of risk-based trading has the potential to reduce the risk of bTB spread [30].

We found also that animals from herds with history of bTB had 3.2 times odds of disease detection compared to herds with no history of bTB. In a tuberculin positive herd which did not remove reactors after skin testing, there could be an increase in infection and hence reactor animals. Even in herds which did cull the reactors, there could be recurrent incidents attributable to persistence of infection in such herds due to failure to detect and remove all infected cattle associated with the performance of the skin test [25].

Exotic and cross bred cattle are known to be more susceptible to bTB [8, 25]. Here we found three times (95% CI: 1.5-5.8) odds of bTB detection in these breeds compared to the indigenous zebu breed. The strategy to meet high milk demand is still geared towards improved dairy cattle as a crossbred dairy cow produces on average at least five times more milk than an indigenous zebu cow [21].With the Ethiopian Government setting a policy to significantly increase the number of crossbred cattle, intensification is likely to increase and thereby the risk of bTB transmission [12, 23].The final important risk factor we identified is the introduction of cattle to

the herd through purchase. We found that cattle purchased from another farm were more often reactors (37.5%) compared to cattle bred at own farm (30%). Although this difference is not statistically significant, it warrants further investigation.

Overall, when comparing our study with previous surveys of dairy cattle in this established dairy belt of Ethiopia, there was no major difference in bTB animal prevalence but our study showed a slight increase in herd prevalence. Firdessa and colleagues [5]recorded in 2009/2010 a 30% (n=2,956) animal and 50% (n=88) herd level bTB prevalence while Tsegaye and colleagues [10]in 2006/2007 recorded 34.1% (n=1,132) animal and 53.6% (n=56) herd bTB prevalence, respectively, which is comparable to our corresponding figures. This consistency over time suggests that bTB has reached an endemic equilibrium in these herds. The burden of bTB in the dairy belt in central Ethiopia (31%) is much greater than for emerging dairies in regional states, estimated to range from 0.3% to 12% animal prevalence [6, 11,13,24]. The central region of the country should thereby be considered as a bTB high risk area and this report opens up for a scientific approach for future risk-based surveillance and disease intervention. Cattle trading from this region pose high risk of introducing bTB infection to new herds and underlines the significance of cattle trade regulation with pre-movement testing. The significantly lower bTB prevalence recorded in many emerging dairies in the regional states (which could be considered as low risk regions) presents an opportunity for intervention*e.g.*by trade restrictions to prevent further disease transmission from high risk areas like central Ethiopia and introduce testing to support farmers to keep their herds free from bTB. A recent survey by Mekonnen and colleagues[6]recorded an average disease rate of 5.2% (95% CI: 4-6%)in three emerging dairies in regional states, including Hawassa (3%), Gondar (1.4%), and Mekelle (12%). An earlier report from 2014 [31]documented also lower prevalence (below 7%) in eight out of twelve emerging

dairies, but ranging from 0.8% to 24% with a few hot spots in Kombolcha (24%) and Mekelle (14%), the latter confirmed by Mekonnen et al.[6].The lower bTB rates in many of these emerging dairy regions could be due to less cattle movement from high risk regions and less intensification, as they may have emergedmore recently.However, if these emerging dairy regions will intensify, and without a strategy for bTB disease control in Ethiopia, it is likelythat these regions will be more affected by bTB in the future.

As a limitation of this study; in some of the herds which lack records-for some of the risk factors such as age, data was collected through interview. People may not recall specially for older animals. We have tried to compliment this age estimation with parity and dentition data.

Conclusions

The present study reported a high level of bTB prevalence in the large dairy belt around the capital Addis Ababa in central Ethiopia based on the SICCT test. High variability in burden of infection among the tested dairy herds was also an important finding of this study as it can have impact on future disease intervention strategies. In addition, it identified herd size, animal age, cattle breed, and bTB history at farm as important risk factors contributing to the high prevalence of bTB in the central parts of the country. As the Ethiopian dairy sector is expanding, especially through emerging new dairies around many other urban centers across the country, the findings from this study add useful epidemiological information critical for the application of targeted evidence-based control measures. Therefore, there is now an opportunity to take steps towards a strategy that can control and/or significantly reduce the burden of bTB in Ethiopia to improve animal and human health.

Supporting information

S1Fig. Map showing the geographical locations and the sizes of bTB-positive and negative herds and within-herd prevalence in central Ethiopia.

S1 Supplementary. Additional information on farm characteristics, description of risk factors and risk factors analysis.

S1 Questionnaire. Questionnaire for collection of epidemiological data of bovine tuberculosis in central Ethiopia.

S1 Dataset(ZIP). Raw data

Conflict of Interest

The authors declare that they have no conflict of interests.

Acknowledgements

This research was financially supported by the Ethiopia Control of Bovine Tuberculosis Strategies (ETHICOBOTS) project funded by the Biotechnology and Biological Sciences Research Council, the Department for International Development, the Economic & Social Research Council, the Medical Research Council, the Natural Environment Research Council and the Defence Science &Technology Laboratory, under the Zoonoses and Emerging Livestock Systems (ZELS) program, ref: BB/L018977/1.Stefan Berg was also funded by Defra, United

Kingdom, ref: TBSE3294. We thank NAHDIC for their logistical support. The authors would like to acknowledge Drs Matios Lakew, Nebyou Kassa,Worku Birhanu, Getachew Tuli and Asamnew Tesfaye, Bekele Yalew, MekedesTamiru, Daniel Tekeste, Chala Dimma, Teferi Benti, Demessa Negessu, all participating dairy farmers, district veterinary officers and institutions for their support during the field work.

The members of the ETHICOBOTSconsortium are: Abraham Aseffa, Adane Mihret, Bamlak Tessema, Bizuneh Belachew, Eshcolewyene Fekadu, Fantanesh Melese, Gizachew Gemechu, Hawult Taye, Rea Tschopp, Shewit Haile, Sosina Ayalew, Tsegaye Hailu, all from Armauer Hansen Research Institute, Ethiopia; Rea Tschopp from Swiss Tropical and Public Health Institute, Switzerland; Adam Bekele, Chilot Yirga, Mulualem Ambaw, Tadele Mamo, Tesfaye Solomon, all from Ethiopian Institute of Agricultural Research, Ethiopia; Tilaye Teklewold from Amhara Regional Agricultural Research Institute, Ethiopia; Solomon Gebre, Getachew Gari, Mesfin Sahle, Abde Aliy, Abebe Olani, Asegedech Sirak, Gizat Almaw, Getnet Mekonnen, Mekdes Tamiru, Sintayehu Guta, all from National Animal Health Diagnostic and Investigation Center, Ethiopia; James Wood, Andrew Conlan, Alan Clarke, all from Cambridge University, United Kingdom; Henrietta L. Moore and Catherine Hodge, both from University College London, United Kingdom; Constance Smith at University of Manchester, United Kingdom; R. Glyn Hewinson, Stefan Berg, Martin Vordermeier, Javier Nunez-Garcia, allfrom Animal and Plant Health Agency, United Kingdom; Gobena Ameni, Berecha Bayissa, Aboma Zewude, Adane Worku, Lemma Terfassa, Mahlet Chanyalew, Temesgen Mohammed, Miserach Zeleke, all from Addis Ababa University, Ethiopia.

Author Contributions

Conceptualization: James LN Wood, Stefan Berg, Gobena Ameni, Adane Mihret

Data curation: Gizat Almaw, Andrew JK Conlan, Demeke Nigussie

Formal analysis: Gizat Almaw, Andrew JK Conlan

Investigation: Gizat Almaw,Alemseged Alemu, Sintayehu Guta,Abebe Olani, Abebe Garoma, Dereje Shegu, Letebrhan Yimesgen, Tafesse Koran, Solomon Gebre

Methodology: Gizat Almaw, James LN Wood, Stefan Berg, Gobena Ameni, Adane Mihret

Project administration: Solomon Gebre, Adane Mihret, Stefan Berg, James LN Wood

Supervision: Adane Mihret, Tamrat Abebe, Gobena Ameni, Balako Gumi, Solomon Gebre, Stefan Berg

Writing – original draft: Gizat Almaw

Writing – review & editing: Gizat Almaw, Stefan Berg, Andrew JK Conlan, Adane Mihret, Tamrat Abebe, Gobena Ameni, Balako Gumi

References

1. OIE.Terrestrial manual: Bovine tuberculosis, Manual of Diagnostic Tests and Vaccines for Terrestrial Animals. 2018. p.1058-1074.

2. Müller B, Dürr S, Alonso S, Hattendorf J, Laisse CJM, Parsons SDC, et al. Zoonotic *Mycobacterium bovis* –induced Tuberculosis in Humans. Emerg Infect Dis. 2013;19:899–908.

3. CSA-Ethiopia. Population Projections 2007-2037. 2012.

4. Ahmed MM, Ehui S, Yemesrach Assefa. Dairy development in Ethiopia. Washington, DC, USA : Nairobi, Kenya: International Food Policy Research Institute ; International Livestock Research Institute; 2003.

5. Firdessa R, Tschopp R, Wubete A, Sombo M, Hailu E, Erenso G, et al. High Prevalence of Bovine Tuberculosis in Dairy Cattle in Central Ethiopia: Implications for the Dairy Industry and Public Health. Langsley G, editor. PLoS ONE. 2012;7:e52851.

6. Mekonnen GA, Conlan AJK, Berg S, Ayele BT, Alemu A, Guta S, et al. Prevalence of bovine tuberculosis and its associated risk factors in the emerging dairy belts of regional cities in Ethiopia. Prev Vet Med. 2019;168:81–89.

7. Ameni G, Amenu K, Tibbo M. Bovine Tuberculosis: Prevalence and risk factor assessment in cattle and cattle owners in Wuchale-Jida District, central Ethiopia. Journal of Applied Research in Veterinary Medicine. 2003; 1:17-26.

8. Vordermeier M, Ameni G, Berg S, Bishop R, Robertson BD, Aseffa A, et al. The influence of cattle breed on susceptibility to bovine tuberculosis in Ethiopia. Comparative Immunology, Microbiology and Infectious Diseases. 2012;35:227–232.

9. Tschopp R, Schelling E, Hattendorf J, Aseffa A, Zinsstag J. Risk factors of bovine tuberculosis in cattle in rural livestock production systems of Ethiopia. Preventive Veterinary Medicine. 2009;89:205-211.

10. Tsegaye W, Aseffa A, Mache A, Mengistu Y. Conventional and Molecular Epidemiology of Bovine Tuberculosis in Dairy Farms in Addis Ababa City, the Capital of Ethiopia.Intern J Appl Res Vet Med. 2010;8:143-151.

11. Dejene SW, Heitkönig IMA, Prins HHT, Lemma FA, Mekonnen DA, Alemu ZE, et al. Risk Factors for Bovine Tuberculosis (bTB) in Cattle in Ethiopia. PLOS ONE. 2016;11:e0159083.

12. Sibhat B, Asmare K, Demissie K, Ayelet G, Mamo G, Ameni G. Bovine Tuberculosis in Ethiopia: a Systematic Review and Meta-analysis. Preventive veterinary medicine. 2017;147:149-157.

13. Tschopp, R., Hattendorf, J., Roth, F., Choudhury, A., Shaw, A., Aseffa, A., Zinsstag, J. Cost estimate of bovine tuberculosis to Ethiopia. Curr. Top. Microbiol. 2012. Immunol. 365, 249–268.

14. Skuce RA, Allen AR, McDowell SWJ. Herd-Level Risk Factors for Bovine Tuberculosis: A Literature Review. In: Veterinary Medicine International. 2012. doi:10.1155/2012/621210

15. Bessell PR, Orton R, White PCL, Hutchings MR, Kao RR. Risk factors for bovine Tuberculosis at the national level in Great Britain. BMC Veterinary Research. 2012;8:51.

16. Thrusfield, M. Veterinary epidemiology. 3rd Edition, Blackwell Science Ltd., Oxford.2007.

17. R Core Team. R A Language and Environment for Statistical Computing. R Foundation for Statistical Computing, Vienna. - References - Scientific Research Publishing. 2018.URL https://www.R-project.org/.

18. Bates D, Mächler M, Bolker B, Walker S. Fitting Linear Mixed-Effects Models using lme4. 2014. Available: http://arxiv.org/abs/1406.5823

19. Sing T, Sander O, Beerenwinkel N, Lengauer T. ROCR: visualizing classifier performance in R. Bioinformatics. 2005;21:3940–3941.

20. Ameni G., AseffaA., Sirak A., EngersH., YoungB. D., Hewinson R.G., Vordermeier H. M. and GordonV. S. Effect of skin testing and segregation on the incidence of bovine tuberculosis, and molecular typing of Mycobacterium bovis in Ethiopia. Vet Rec. 2007; 161:782–786.

21. Tegegne A, Gebremedhin B, Hoekstra D, Belay B, Mekasha Y. Smallholder dairy production and marketing systems in Ethiopia: IPMS experiences and opportunities for market-

oriented development. IPMS (Improving Productivity and Market Success) of Ethiopian Farmers Project Working Paper 31. Nairobi: ILRI.

22. Shapiro BI, Gebru G, Desta S, Negassa A, Negussie K, Aboset G, et al. Ethiopia livestock master plan: Roadmaps for growth and transformation. Ministry of Agriculture and ILRI; 2015.p.35-51.

23. Ameni G, Aseffa A, Engers H, Young D, Hewinson G, Vordermeier M. Cattle Husbandry in Ethiopia Is a Predominant Factor Affecting the Pathology of Bovine Tuberculosis and Gamma Interferon Responses to Mycobacterial Antigens. Clinical and Vaccine Immunology. 2006;13:1030–1036.

24. Habitu T, Areda D, Muwonge A, Tessema GT, Skjerve E, Gebrehiwot T. Prevalence and risk factors analysis of bovine tuberculosis in cattle raised in mixed crop-livestock farming system in Tigray region, Ethiopia. Transboundary and Emerging Diseases. 2019;66:488–496.

25. Broughan JM, Judge J, Ely E, Delahay RJ, Wilson G, Clifton-Hadley RS, et al. A review of risk factors for bovine tuberculosis infection in cattle in the UK and Ireland. Epidemiol. Infect. 2016; 144:2899–2926.

26. Humblet M-F, Boschiroli ML, Saegerman C. Classification of worldwide bovine tuberculosis risk factors in cattle: a stratified approach. Vet Res. 2009;40:50.

27. Clegg TA, Good M, Hayes M, Duignan A, McGrath G, More SJ. Trends and Predictors of Large Tuberculosis Episodes in Cattle Herds in Ireland. Front Vet Sci. 2018;5.

28. Pascual-Linaza AV, Gordon AW, Stringer LA, Menzies FD. Efficiency of slaughterhouse surveillance for the detection of bovine tuberculosis in cattle in Northern Ireland. Epidemiol Infect. 2017;145:995–1005.

29. McKinley TJ, Lipschutz-Powell D, Mitchell AP, Wood JLN, Conlan AJK. Risk factors and variations in detection of new bovine tuberculosis breakdowns via slaughterhouse surveillance in Great Britain.PLoS ONE. 2018; 13(6):e0198760.

30. Adkin A, Brouwer A, Downs SH, Kelly L. Assessing the impact of a cattle risk-based trading scheme on the movement of bovine tuberculosis infected animals in England and Wales. Prev Vet Med. 2016;123:23–31.

31. Alehegne, W., Gizat, A., Tafesse, K., Mekedes, T., Mesfin, S., Getnet, A. et al. Bovine Tuberculosis Study in Farmed Dairy Cattle and Cattle Genetic Improvement Centers in Selected Areas of Ethiopia. Abstract at the Mbovis 2014 conference June 2014. Cardiff, Wales. 2014.

32. Dohoo L., Martin W., Stryhn H. Veterinary Epidemiologic Research. 2003.

33. Conlan A. J. K., McKinley T. J., Karolemeas K., Brooks Pollock E. , Goodchild A. V., Mitchell A. P., Birch C. P. D., Clifton-Hadley R. S., Wood J.L. N. Estimating the Hidden Burden of Bovine Tuberculosis in Great.PLoS Comput Biol 8(10):2012. e1002730.

34. NAHDIC. Test Result Report-NAHDIC-FRM-GEN-025-2A(2011 to 2014)

Figure

Click here to access/download;Figure;Fig 2.TIF.TIF

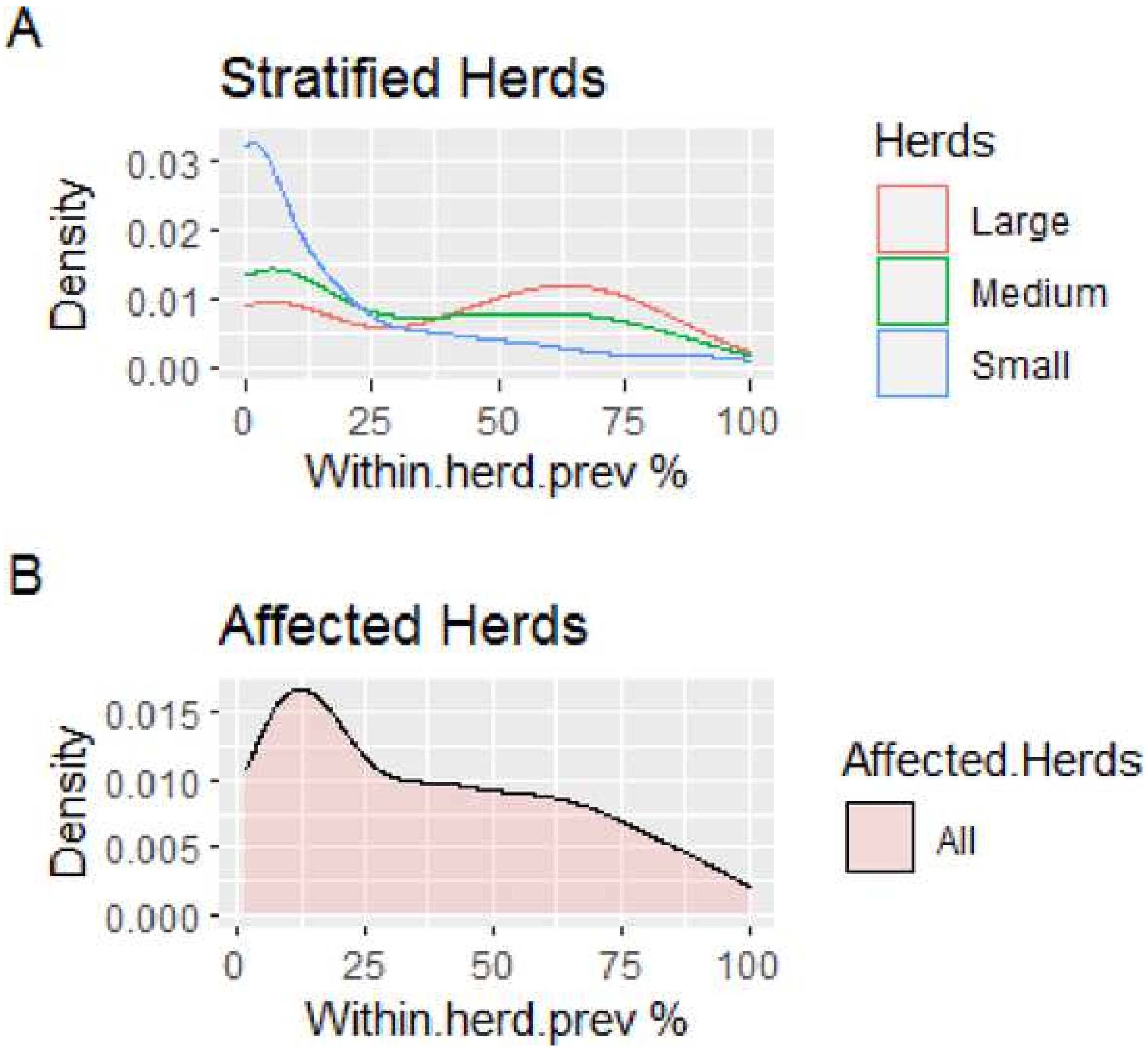

Figure

Click here to access/download;Figure;Fig1.tif.tif

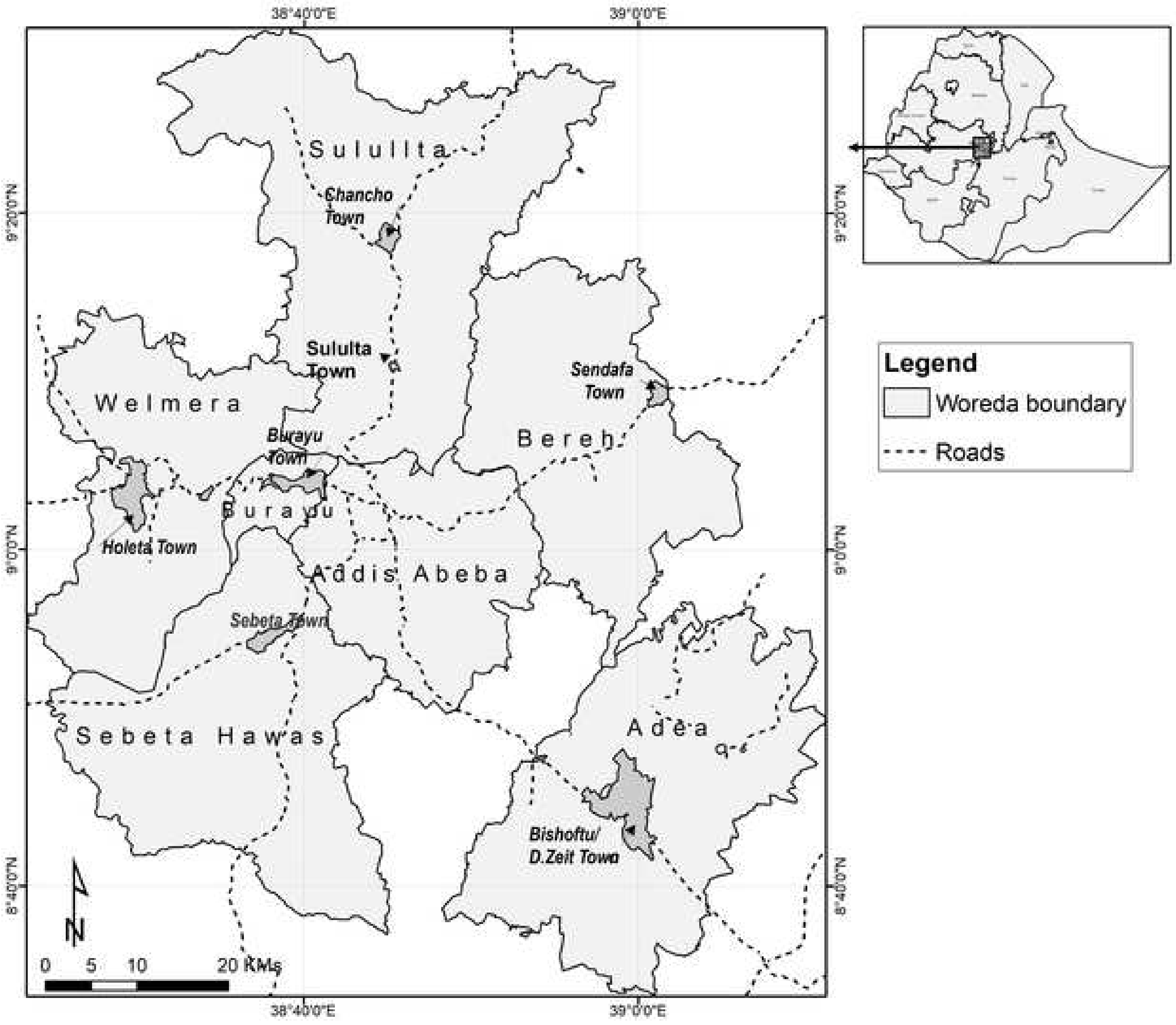

Click here to access/download
Supporting Information - Compressed/ZIP File Archive
S 1 Supplementary.doc

Click here to access/download
Supporting Information - Compressed/ZIP File Archive
S1 Dataset Raw Data.csv

Click here to access/download
Supporting Information - Compressed/ZIP File Archive
S1 Questionnaire.doc

Click here to access/download
Supporting Information - Compressed/ZIP File Archive
S1_Fig.tif.tif

Below we present a summarized response to the reviewers' comments.

The major criticism seemed to have been the lack of significant contribution to the field, which I understand to be a specific criterion stated as not used by PLoS ONE in its decision making.

The most significant concern seemed to be:

- "The knowledge gap is not clear. What new insights are the author intending to reveal to persuade the readers the novelty of this work other than imitating what have been done previously?"

Response: We believe there was enough reason to undertake this study (irrespective of stated PLoS ONE policy). Our paper confirms and updates the importance of bTB as a problem in central Ethiopia. The extent of the problem was not quantified contemporaneously to all for specific control strategies. No other study covering the area attempted a one stage cluster sampling, and none sampled more than 100 farms, the most recent survey being in 2008/9. In the revised version we have also demonstrated differences in within herd prevalence of bTB (stratified by herd size), important information for future design of control strategies. In addition, we have improved understanding of the risk factors among dairy farms in Ethiopia.

Besides this major concern, there were a number of technical queries including

- "Sample size calculation - Using the formula and data you provided it gives 393.6 herds required for the study. But 299 herds were used in your study. Can you please clarify that?"

Response: It was adjusted for a finite or small population to 298, as described in the paper. The number of 394 represented calculations for an infinite population. Our sampling frame was finite so it was appropriate to adjust the sample size to 298 adjusting for the population size; 299 herds were tested.

- It is not clear why you decided to have 212:44:43 for the small, medium and large herd categories? Is this percentage (212:44:43) representative to the distribution of the herds in the study areas?

Response: Thank you. We have revisited this following the reviewer comment. The fractions in the study sample were 71%, 16% and 13% and those in the overall population were 89%, 7% and 4%. The over-representation of larger farms was due to a greater level of refusal to participate in smaller herds, despite efforts to address this, and numbers were made up in medium and larger herds. We used a direct standardization approach to explore what the impact of this was and found that the overall animal level prevalence changed from 31.3% to 24.5%, and the corresponding herd level prevalence changed from 60.9% to 54.4%. We have adjusted our manuscript accordingly. This standardisation provides better estimates of true bTB prevalence in central Ethiopia and has not been used in other studies, highlighting again the relevance of this study.

- Statistical analysis: The comments raised by reviewers include: Could you please add the formula of the models that used in the analysis?, Why you did not use the region as a random effect in your model?, What was your criteria for building your multivariate model?, are these the only risk factors that you tested in your models?, It is recommended that the authors use mathematical expression to help readers identify which model was used., Not so sure why the

authors– chose to ignore the use of univariable analysis first to assess effects of individual variables on outcome variable.

Response: we have addressed these issues. Using our large data set allowed more (16) potential risk factors to be considered, as per reviewer's comment. Our dataset was hierarchal in nature *i.e*. individual animals were clustered within herds and herds were clustered within study areas. To account for this clustering and deal with variation in prevalence between study areas and in particular between herds, Generalized Linear Mixed Model (GLMM) was used which allowed us to treat herd and study areas as random effects with a binary outcome variable (bTB reactor or not reactor) at the animal level. We used the glmer() function in the lme4 package. Univariable screening ($p < 0.20$) was used to select variables for inclusion in the multivariable model. Candidate explanatory variables were investigated for collinearity requiring that all selected variables for the multivariable model had a variance inflation factor (VIF) of < 5.

- The reviewer expressed concern that not all data underlying the findings were fully available.

  Response: All raw data are fully available.

  - Do you have IRB approval for the survey?

  Response: yes and we have included it in the revised manuscript.

Given that we have addressed the issues raised by reviewers and that lack of significance is stated as an irrelevant consideration for PLoS ONE, I would be very grateful if you would reconsider this manuscript (with its changed title as per reviewer's comment) for publication. Thank you very much in advance for your consideration.

Best wishes

Gizat Almaw (corresponding author)
Address:
National Animal Health Diagnostic and Investigation Center, Sebeta, Ethiopia & Department of Microbiology, Immunology and Parasitology, College of Health Sciences, Addis Abeba University, Addis Abeba, Ethiopia
E-mail: gizatalm@yahoo.com
Mob Phone: + 251960080833

PONE-D-19-32366
Uncontrolled bovine tuberculosis remains a challenge for dairy development and public health in central Ethiopia
PLOS ONE

Dear Dr Almaw,

Thank you for submitting your manuscript to PLOS ONE. After careful consideration, we have decided that your manuscript does not meet our criteria for publication and must therefore be rejected.

Your manuscript has been reviewed by two experts in the field. Both have raised important concerns regarding the study design, statistical analysis, and data interpretation. I concur with their view. Unfortunately, these concerns are of sufficient entity to preclude considering this manuscript further.

I am sorry that we cannot be more positive on this occasion, but hope that you appreciate the reasons for this decision.

Yours sincerely,

Angel Abuelo, DVM, MRes, MSc, PhD, DABVP (Dairy), DECBHM
Academic Editor
PLOS ONE

[Note: HTML markup is below. Please do not edit.]

Reviewers' comments:

Reviewer's Responses to Questions

Comments to the Author

1. Is the manuscript technically sound, and do the data support the conclusions?

The manuscript must describe a technically sound piece of scientific research with data that supports the conclusions. Experiments must have been conducted rigorously, with appropriate controls, replication, and sample sizes. The conclusions must be drawn appropriately based on the data presented.

Reviewer #1: Partly

Reviewer #2: Partly

2. Has the statistical analysis been performed appropriately and rigorously?

Reviewer #1: N/A

Reviewer #2: No

---

3. Have the authors made all data underlying the findings in their manuscript fully available?

The PLOS Data policy requires authors to make all data underlying the findings described in their manuscript fully available without restriction, with rare exception (please refer to the Data Availability Statement in the manuscript PDF file). The data should be provided as part of the manuscript or its supporting information, or deposited to a public repository. For example, in addition to summary statistics, the data points behind means, medians and variance measures should be available. If there are restrictions on publicly sharing data—e.g. participant privacy or use of data from a third party—those must be specified.

Reviewer #1: No

Reviewer #2: No

4. Is the manuscript presented in an intelligible fashion and written in standard English?

PLOS ONE does not copyedit accepted manuscripts, so the language in submitted articles must be clear, correct, and unambiguous. Any typographical or grammatical errors should be corrected at revision, so please note any specific errors here.

Reviewer #1: Yes

Reviewer #2: Yes

---

5. Review Comments to the Author

Please use the space provided to explain your answers to the questions above. You may also include additional comments for the author, including concerns about dual publication, research ethics, or publication ethics. (Please upload your review as an attachment if it exceeds 20,000 characters)

Reviewer #1: The submitted article addresses the prevalence of bovine tuberculosis which

considered as one of the major problems affecting the dairy industry and attract the attention of the dairy producers and public health professionals. The study has been done on large number of herds (299) covering six different geographical areas in Central Ethiopia. The study used comparative intradermal skin test for diagnosis of bovine tuberculosis. Although overall the paper has merit but there are number of issues that I would like the authors to consider which I hope will improve the paper. The manuscript requires major modifications in its style, presentation of the results and written format to make it clear to the reader.

Not all the supporting information are available. Please add the raw data and a copy of the survey questions.

***Response**: All supporting information including raw data and a copy of the survey questions are included in the revised Manuscript.*

1- Abstract:
- Number of animals included in the study are not consistent along the abstract, materials and methods and results. For example: Total number of animals tested according to the herd size in table 2 are 5679 (2061+1038+2580= 5679) while in your abstract (n=5675, line 30), materials and methods (5675, line 116), results (5,675, line 168). Please clarify

***Response**: comment accepted. This is a mistake. The four animal difference is ONLY in table 2 and the remaining section is consistent total 5675 animals across as stated by the reviewer also. These 4 animals were those with no test result (sold during in the 2nd round reading). And shown here under as* # VALUE!:4.

```
Result
 #VALUE!:   4
 FALSE  :3899
 TRUE   :1776
```

However, this will not affect our analysis for risk factors as this was taken care of by the under r code. R treat this as missing value (NA).

Allregion$Result[which(Allregion$Result=='#VALUE!')]=NA

2- Introduction:
- The knowledge gap is not clear

> ***Response**: We have tried to show this in the revised manuscript. Our paper confirms and updates the importance of bTB as a problem in central Ethiopia. The extent of the problem was not quantified contemporaneously to all for specific control strategies. No other study covering the area attempted a one stage cluster sampling, and none sampled more than 100 farms, the most recent survey being in 2008/9. In the revised version we have also demonstrated differences in within herd prevalence of bTB (stratified by herd size), important information for future design of control strategies. In*

addition, we have improved understanding of the risk factors among dairy farms in Ethiopia.

- There is no research hypothesis.

Response *: Our hypothesis was the current extent of bTB (stratified by herd size) and associated risk factors was not known in central Ethiopia.*

3- Material and methods:
- What are the inclusion and exclusion criteria for the study herds?

Response*: Herd size was the criteria used and herds with less than five animals were excluded and this is mentioned in the revised manuscript.*

- Sample size calculation:
o Using the formula and data you provided it gives 393.6 herds required for the study. But 299 herds were used in your study. Can you please clarify that?

Response: *All is described in detail in the revised paper. It was adjusted for a finite or small population to 298. The number of 393.6 represented calculations for an infinite population. Our sampling frame was finite so it was appropriate to adjust the sample size to 298 adjusting for the population size; 299 herds were tested. 393.6 herds number was obtained from the formula (*$g = \frac{1.96^2\{nVC+Pexp(1-Pexp)\}}{nd^2}$*,) which assumes for infinite/large population.*

$g_{adj} = \frac{G*g}{G+g}$*, G=total number of herds & g=is the calculated sample size for infinite/large herd*

$$g_{adj} = \frac{1323 * 383}{1323 + 383} = 297.01582649472 = 298\ herds$$

We have rounded variance between herds Vc = 0.232903692614545 (actual value we obtained) to Vc=0.24 and this increased number of herds from 383 to 393.6(calculated by the reviewer). Vc = 0.233 is used in the revised paper and when fitted to the formula gives 383 herds. And we used this 383 for sample adjustment as can be seen in the above formula.

o It is not clear why did you decide to have 212:44:43 for the small, medium and large herds categories? Is this percentage (212:44:43) representative to the distribution of the herds in the study areas?

Response: *Thank you. We have revisited this following the reviewer comment. Our approach was proportional allocation where the number of herds selected were proportional to the number in each herd category. The fractions in the study sample were 71%, 16% and 13% and those in the overall population were 89%, 7% and 4%. The over-representation of larger farms was due to a greater level*

of refusal to participate in smaller herds, despite efforts to address this, and numbers were made up in medium and larger herds. We used a direct standardization approach to explore what the impact of this was and found that the overall animal level prevalence changed from 31.3% to 24.5%, and the corresponding herd level prevalence changed from 60.9% to 54.4%. We have adjusted our manuscript accordingly. This standardisation provides better estimates of true bTB prevalence in central Ethiopia and has not been used in other studies, highlighting again the relevance of this study.

o It is not clear how did you decide on the number of animals to be tested by CIDT at the herd level? Do you mean that all animals in the 299 herds were tested excluding animals less than 3 months of age and pregnant cattle ≥8 months pregnant are 5675?

Response: *Yes ! stated in the paper as " Sample size was determined following one-stage cluster sampling method taking dairy herd as a cluster [16] and **every animal in the selected cluster was tested**."*

- Comparative intradermal tuberculin test:

o Who did and interpret the test results?

Response: *the same person(experienced technician). It is stated in the paper as" The two injection sites were re-measured after 72 hours **by the same person** who measured the skin thickness before the injection." line 134 & 135.*

o How did you choose the animals to be tested from each herd?

Response: *It was the herds that were selected randomly and all animals fulfilling the inclusion criteria in the selected herd were tested. And this has been mentioned in the paper.*

- Epidemiological data collection:

o Do you have IRB approval for the survey?

Response*: Yes and is included in the revised paper.*

- Statistical analysis:

o In the Analysis GLMM was used:

Did you model the risk factors at the animal level or at the herd level?♣

Response: *at animal level and this has been mentioned in the paper.*

What is your outcome?♣

Response: *binary response as an outcome variable (bTB reactor or not reactor).*

Could you please add the formula of the models that used in the analysis?♣

Response: *Accepted! And the formula is included in the revised manuscript.*

Why you did not use the region as a random effect in your model?♣

Response: *Now included in the revised data analysis and included in the revised paper also which reads as* **"** Our dataset was hierarchal in nature *i.e.* individual animals were clustered within herds and herds were clustered within study areas. To account for this clustering and deal with variation in prevalence between study areas and in particular between herds, Generalized Linear Mixed Model (GLMM)[32]was used which allowed us to treat herd and study areas as random effects with a binary response as an outcome variable (bTB reactor or not reactor)."

What was your criteria for building your multivariate model?♣

Response*: All variables with a p-value of < 0.20 and those with a high biological relevance were considered as candidate variables for the model building.*

4- Results: - I did not see any data in the results about the descriptions of the demographics of the enrolled herds (299). Please add these data if you have it.

Response: *addressed in the revised paper see Table 1* **"**Table 1. Herd structure of the 299 studied

dairy herds."

- Line 174: “There was significant variation between study areas in prevalence of tuberculin reactors” why you did not account for that variation in your multivariate model?

Response*: explained above*

- Line 189: “Eight potential risk factors were analyzed using a GLMM with herd as a random effect” are these the only risk factors that you tested in your models?

Response*: Our large data set allowed more (16) potential risk factors to be considered, as per reviewer’s comment. Hence eight more risk factors are included in the revised paper.*

- Data in table 2 are confusing :

o Data in columns named levels, number of cattle tested and btB reactors (%) are confusing and need to be corrected.

***Response**: Addressed. See Table 4 in the revised paper.*

For example: Herd size♣ categories (4,20), (20,35) and (35,168) Did you include 20 in both small and medium sized herds counts? The same for 35.

***Response**: Not. in small it is bracket "]" means- it includes and in medium it is parenthesis "(" means- it does not include. In the revised paper we have included clarification for this as a foot note.*

Total number of♣ animals according to the herd size are 5679 (2061+1038+2580= 5679) while in your abstract (n=5675, line 30), materials and methods (5675, line 116), results (5,675, line 168), which one is correct? Please clarify.

***Response:** addressed in the preceding section.*

Age categories (0.2,2), (2,4), (4,6), (6,8) (8,10) the same comment as the herd size. Please clarify.♣

***Response**: Explained above.*

Total number of animals according to the age categories are 5260 (1981+1422+1096+564+197= 5260) while in your abstract (n=5675, line 30), materials and methods (5675, line 116), results (5,675, line 168), which one is correct? Please clarify.

***Response:** Accepted and corrected. General comment-number of cattle for descriptive and analytical analysis may differ due to missing values. This was the case for table 2. Total animals for age analysis are 5256 & the remaining 419 are missing values but this data can be used for prevalence estimation in the descriptive section.*

Source, breed, sex, farm age, btB history, and animal contact. (same comment as the herd size and age).

***Response**: checked and corrected*

The number of animals in the bTB reactors (%) column (same comment as the column of number of tested cattle)♣

***Response:** checked.*

- Why did you include non-significant variables in table 2? For example, sex, source, farm age and animal contact.

***Response:** addressed. Now in the revised paper, divided in to univariable screening and final model, the later included only significant ones.*

- What is your explanation for the wide 95% CI for most of the variables that included in the table? For instance, large herd size 95% CI is 8.37, 152.72. did you test for other confounders and effect modifiers? Please explain.

Response: *this could be due large variation between farms.*

5-Discussion- Lines 251:255, What are these risk factors?

Response: *cattle movement policy may differ between regions; feeding, housing, bio-security issues do vary between regions/countries.*

- What are the limitations of your study?

Response: *Addressed in the revised paper.* **"** As a limitation of this study; in some of the herds which lack records-for some of the risk factors such as age, data was collected through interview. People may not recall specially for older animals. We have tried to compliment this age estimation with parity and dentition data."

Reviewer #2 The title "Uncontrolled bovine tuberculosis remains a challenge–: for dairy development and public health in central Ethiopia" is misleading. What evidences did the study provide regarding the impacts the infection has caused on public health and socioeconomics other than describing the results of preliminary prevalence study? It is recommended that title be modified to precisely reflect the work done.

Response: *accepted and title modified in the revised paper.*

There are– plenty of prevalence studies carried out on BTB in Ethiopia including the central region where this study was carried out (as the author themselves indicated). So, what new insights are the author intending to reveal to persuade the readers the novelty of this work other than imitating what have been done previously? It would have been more appropriate to apportion the resource used to conduct this study toward pilot study for BTB control and prevention.

Response: *We believe there was enough reason to undertake this study (irrespective of stated PLoS ONE policy). Our paper confirms and updates the importance of bTB as a problem in central Ethiopia. The extent of the problem was not quantified contemporaneously to all for specific control strategies. No other study covering the area attempted a one stage cluster sampling, and none sampled more than 100 farms, the most recent survey being in 2008/9. In the revised version we have also demonstrated differences in within herd prevalence of bTB (stratified by herd size), important information for future design of control strategies. In addition, we have improved understanding of the risk factors among dairy farms in Ethiopia.*

There seems to be a– hierarchical (multi-level) structure of data (study area>>herd>>>individual animals. The use of GLMM to account for infection clustering at various levels is, therefore, appropriate. However, it is recommended that the authors use mathematical expression to help readers identify which model was used (linear regression, logistic regression, and Poisson regression?). Understandably, logistic model is fitting since the outcome variable is of binary type. Nevertheless, this has to be explained to the readers explicitly.

Response: *Accepted. And mathematical expression is included in the revised paper. In the revised paper, detailed description is provided in the statistical analysis section.*

Not so sure why the authors– chose to ignore the use of univariable analysis first to assess effects of individual variables on outcome variable. Understandably, relatively fewer variables were recorded in the study but still plausible to assess their individual and interactive effects on outcomevariable.

Response: *Univariable analysis is included in the revised paper (Table 4). Our large data set allowed more (16) potential risk factors to be considered. We have analyzed by including additional risk factors.*

– Line 80 “Six study areas were selected in the urban areas of central Ethiopia”. The authors did not mention how these areas were selected. Was it random or systematic/purposeful?

Response: *The selection of these study areas was purposeful. The study areas are currently the main milk suppliers for people in Addis Ababa and the surrounding peri-urban areas. This description is included in the revised paper.*

---

6. PLOS authors have the option to publish the peer review history of their article (what does this mean?). If published, this will include your full peer review and any attached files.
If you choose “no”, your identity will remain anonymous but your review may still be made public.
Response: *May be we do not understand it well during online submission. We choose "Yes" for the revised paper.*
[NOTE: If reviewer comments were submitted as an attachment file, they will be attached to this email and accessible via the submission site. Please log into your account, locate the manuscript record, and check for the action link "View Attachments". If this link does not appear, there are no attachment files to be viewed.]

- - - - -
For journal use only: PONEDEC3
