# Supplementary material for: The variable prevalence of bovine tuberculosis among dairy herds in Central Ethiopia provides opportunities for targeted intervention
Source: PLoS One. 2021 Jul 2;16(7):e0254091. doi: 10.1371/journal.pone.0254091 (PMC8253440; doi:10.1371/journal.pone.0254091)
Supplement: Attachment — Submitted filename: Response to Reviewers.docx [file pone.0254091.s008.docx]

PONE-D-19-32366
Uncontrolled bovine tuberculosis remains a challenge for dairy development and public health in central Ethiopia
PLOS ONE

Dear Dr Almaw,

Thank you for submitting your manuscript to PLOS ONE. After careful consideration, we have decided that your manuscript does not meet our criteria for publication and must therefore be rejected.

Your manuscript has been reviewed by two experts in the field. Both have raised important concerns regarding the study design, statistical analysis, and data interpretation. I concur with their view. Unfortunately, these concerns are of sufficient entity to preclude considering this manuscript further.

I am sorry that we cannot be more positive on this occasion, but hope that you appreciate the reasons for this decision.

Yours sincerely,

Angel Abuelo, DVM, MRes, MSc, PhD, DABVP (Dairy), DECBHM
Academic Editor
PLOS ONE


[Note: HTML markup is below. Please do not edit.]

Reviewers' comments:

Reviewer's Responses to Questions

**Comments to the Author**

1. Is the manuscript technically sound, and do the data support the conclusions?

The manuscript must describe a technically sound piece of scientific research with data that supports the conclusions. Experiments must have been conducted rigorously, with appropriate controls, replication, and sample sizes. The conclusions must be drawn appropriately based on the data presented.

Reviewer #1: Partly

Reviewer #2: Partly

2. Has the statistical analysis been performed appropriately and rigorously?

Reviewer #1: N/A

Reviewer #2: No

3. Have the authors made all data underlying the findings in their manuscript fully available?

The [PLOS Data policy](http://www.plosone.org/static/policies.action#sharing) requires authors to make all data underlying the findings described in their manuscript fully available without restriction, with rare exception (please refer to the Data Availability Statement in the manuscript PDF file). The data should be provided as part of the manuscript or its supporting information, or deposited to a public repository. For example, in addition to summary statistics, the data points behind means, medians and variance measures should be available. If there are restrictions on publicly sharing data—e.g. participant privacy or use of data from a third party—those must be specified.

Reviewer #1: No

Reviewer #2: No

4. Is the manuscript presented in an intelligible fashion and written in standard English?

PLOS ONE does not copyedit accepted manuscripts, so the language in submitted articles must be clear, correct, and unambiguous. Any typographical or grammatical errors should be corrected at revision, so please note any specific errors here.

Reviewer #1: Yes

Reviewer #2: Yes

5. Review Comments to the Author

Please use the space provided to explain your answers to the questions above. You may also include additional comments for the author, including concerns about dual publication, research ethics, or publication ethics. (Please upload your review as an attachment if it exceeds 20,000 characters)

Reviewer #1: The submitted article addresses the prevalence of bovine tuberculosis which considered as one of the major problems affecting the dairy industry and attract the attention of the dairy producers and public health professionals. The study has been done on large number of herds (299) covering six different geographical areas in Central Ethiopia. The study used comparative intradermal skin test for diagnosis of bovine tuberculosis. Although overall the paper has merit but there are number of issues that I would like the authors to consider which I hope will improve the paper. The manuscript requires major modifications in its style, presentation of the results and written format to make it clear to the reader.

Not all the supporting information are available. Please add the raw data and a copy of the survey questions.

***Response****: All supporting information including raw data and a copy of the survey questions are included in the revised Manuscript.*

1- Abstract:
- Number of animals included in the study are not consistent along the abstract, materials and methods and results. For example: Total number of animals tested according to the herd size in table 2 are 5679 (2061+1038+2580= 5679) while in your abstract (n=5675, line 30), materials and methods (5675, line 116), results (5,675, line 168). Please clarify

***Response****: comment accepted. This is a mistake. The four animal difference is ONLY in table 2 and the remaining section is consistent total 5675 animals across as stated by the reviewer also. These 4 animals were those with no test result (sold during in the 2nd round reading). And shown here under as* # VALUE!:4.

```
Result
 #VALUE!:   4
 FALSE  :3899
 TRUE   :1776
```

*However, this will not affect our analysis for risk factors as this was taken care of by the under r code. R treat this as missing value (NA).*

*Allregion$Result[which(Allregion$Result=='#VALUE!')]=NA*

2- Introduction:
- The knowledge gap is not clear

***Response****:* *We have tried to show this in the revised manuscript.* *Our paper confirms and updates the importance of bTB as a problem in central Ethiopia. The extent of the problem was not quantified contemporaneously to all for specific control strategies. No other study covering the area attempted a one stage cluster sampling, and none sampled more than 100 farms, the most recent survey being in 2008/9. In the revised version we have also demonstrated differences in within herd prevalence of bTB (stratified by herd size), important information for future design of control strategies. In addition, we have improved understanding of the risk factors among dairy farms in Ethiopia.*

- There is no research hypothesis.

***Response*** *: Our hypothesis was the current extent of bTB (stratified by herd size) and associated risk factors was not known in central Ethiopia.*

3- Material and methods:
- What are the inclusion and exclusion criteria for the study herds?

***Response****: Herd size was the criteria used and herds with less than five animals were excluded and this is mentioned in the revised manuscript.*

- Sample size calculation:
o Using the formula and data you provided it gives 393.6 herds required for the study. But 299 herds were used in your study. Can you please clarify that?

***Response:*** *All is described in detail in the revised paper.* *It was adjusted for a finite or small population to 298. The number of 393.6 represented calculations for an infinite population. Our sampling frame was finite so it was appropriate to adjust the sample size to 298 adjusting for the population size; 299 herds were tested. 393.6 herds number was obtained from the formula (*$g=\frac{1.96^{2}\left\{ nVC+Pexp(1-Pexp) \right\}}{{nd}^{2}}$,) *which assumes for infinite/large population.*

$g_{adj=}\frac{G*g}{G+g}$*, G=total number of herds & g=is the calculated sample size for infinite/large herd*

$$g_{adj=}\frac{1323*383}{1323+383}=297.01582649472=298 herds$$

*We have rounded variance between herds Vc = 0.232903692614545 (actual value we obtained) to Vc=0.24 and this increased number of herds from 383 to 393.6(calculated by the reviewer). Vc = 0.233 is used in the revised paper and when fitted to the formula gives 383 herds. And we used this 383 for sample adjustment as can be seen in the above formula.*

o It is not clear why did you decide to have 212:44:43 for the small, medium and large herds categories? Is this percentage (212:44:43) representative to the distribution of the herds in the study areas?

***Response:*** *Thank you. We have revisited this following the reviewer comment. Our approach was proportional allocation where the number of herds selected were proportional to the number in each herd category. The fractions in the study sample were 71%, 16% and 13% and those in the overall population were 89%, 7% and 4%. The over-representation of larger farms was due to a greater level of refusal to participate in smaller herds, despite efforts to address this, and numbers were made up in medium and larger herds. We used a direct standardization approach to explore what the impact of this was and found that the overall animal level prevalence changed from 31.3% to 24.5%, and the corresponding herd level prevalence changed from 60.9% to 54.4%. We have adjusted our manuscript accordingly. This standardisation provides better estimates of true bTB prevalence in central Ethiopia and has not been used in other studies, highlighting again the relevance of this study.*

o It is not clear how did you decide on the number of animals to be tested by CIDT at the herd level? Do you mean that all animals in the 299 herds were tested excluding animals less than 3 months of age and pregnant cattle ≥8 months pregnant are 5675?

***Response:*** *Yes ! stated in the paper as " Sample size was determined following one-stage cluster sampling method taking dairy herd as a cluster [16] and* ***every animal in the selected cluster was tested****."*

- Comparative intradermal tuberculin test:

o Who did and interpret the test results?

***Response:*** *the same person(experienced technician). It is stated in the paper as" The two injection sites were re-measured after 72 hours* ***by the same person*** *who measured the skin thickness before the injection." line 134 & 135.*

o How did you choose the animals to be tested from each herd?

***Response:*** *It was the herds that were selected randomly and all animals fulfilling the inclusion criteria in the selected herd were tested. And this has been mentioned in the paper.*

- Epidemiological data collection:

o Do you have IRB approval for the survey?

***Response****: Yes and is included in the revised paper.*

- Statistical analysis:

o In the Analysis GLMM was used:

Did you model the risk factors at the animal level or at the herd level?♣

***Response:*** *at animal level* *and this has been mentioned in the paper.*

What is your outcome?♣

***Response:*** *binary response as an outcome variable (bTB reactor or not reactor).*

Could you please add the formula of the models that used in the analysis?♣

***Response:*** *Accepted! And the formula is included in the revised manuscript.*

Why you did not use the region as a random effect in your model?♣

***Response:*** *Now included in the revised data analysis and included in the revised paper also which reads as* ***"*** Our dataset was hierarchal in nature *i.e*. individual animals were clustered within herds and herds were clustered within study areas. To account for this clustering and deal with variation in prevalence between study areas and in particular between herds, Generalized Linear Mixed Model (GLMM)[32]was used which allowed us to treat herd and study areas as random effects with a binary response as an outcome variable (bTB reactor or not reactor)."

What was your criteria for building your multivariate model?♣

***Response****: All variables with a p-value of < 0.20 and those with a high biological relevance were considered as candidate variables for the model building.*

4- Results: - I did not see any data in the results about the descriptions of the demographics of the enrolled herds (299). Please add these data if you have it.

***Response:*** *addressed in the revised paper see Table 1* ***"***Table 1. Herd structure of the 299 studied dairy herds."

- Line 174: “There was significant variation between study areas in prevalence of tuberculin reactors” why you did not account for that variation in your multivariate model?

***Response****: explained above*

- Line 189: “Eight potential risk factors were analyzed using a GLMM with herd as a random effect” are these the only risk factors that you tested in your models?

***Response****: Our large data set allowed more (16) potential risk factors to be considered, as per reviewer’s comment. Hence eight more risk factors are included in the revised paper.*

- Data in table 2 are confusing :

o Data in columns named levels, number of cattle tested and btB reactors (%) are confusing and need to be corrected.

***Response****: Addressed. See Table 4 in the revised paper.*

For example: Herd size♣ categories (4,20), (20,35) and (35,168) Did you include 20 in both small and medium sized herds counts? The same for 35.

***Response****: Not. in small it is bracket "]" means- it includes and in medium it is parenthesis "(" means- it does not include. In the revised paper we have included clarification for this as a foot note.*

Total number of♣ animals according to the herd size are 5679 (2061+1038+2580= 5679) while in your abstract (n=5675, line 30), materials and methods (5675, line 116), results (5,675, line 168), which one is correct? Please clarify.

***Response:*** *addressed in the preceding section.*

Age categories (0.2,2), (2,4), (4,6), (6,8) (8,10) the same comment as the herd size. Please clarify.♣

***Response****: Explained above.*

Total number of animals according to the age categories are 5260 (1981+1422+1096+564+197= 5260) while in your abstract (n=5675, line 30), materials and methods (5675, line 116), results (5,675, line 168), which one is correct? Please clarify.

***Response:*** *Accepted and corrected. General comment-number of cattle for descriptive and analytical analysis may differ due to missing values. This was the case for table 2. Total animals for age analysis are 5256 & the remaining 419 are missing values but this data can be used for prevalence estimation in the descriptive section****.***

Source, breed, sex, farm age, btB history, and animal contact. (same comment as the herd size and age).

***Response****: checked and corrected*

The number of animals in the bTB reactors (%) column (same comment as the column of number of tested cattle)♣

***Response:*** *checked.*

- Why did you include non-significant variables in table 2? For example, sex, source, farm age and animal contact.

***Response:*** *addressed. Now in the revised paper, divided in to univariable screening and final model, the later included only significant ones****.***

- What is your explanation for the wide 95% CI for most of the variables that included in the table? For instance, large herd size 95% CI is 8.37, 152.72. did you test for other confounders and effect modifiers? Please explain.

***Response:*** *this could be due large variation between farms.*

5-Discussion- Lines 251:255, What are these risk factors?

***Response:*** *cattle movement policy may differ between regions; feeding, housing, bio-security issues do vary between regions/countries.*

- What are the limitations of your study?

***Response:*** *Addressed in the revised paper****. "*** As a limitation of this study; in some of the herds which lack records-for some of the risk factors such as age, data was collected through interview. People may not recall specially for older animals. We have tried to compliment this age estimation with parity and dentition data."

Reviewer #2 The title “Uncontrolled bovine tuberculosis remains a challenge−: for dairy development and public health in central Ethiopia” is misleading. What evidences did the study provide regarding the impacts the infection has caused on public health and socioeconomics other than describing the results of preliminary prevalence study? It is recommended that title be modified to precisely reflect the work done.

***Response:*** *accepted and title modified in the revised paper.*

There are− plenty of prevalence studies carried out on BTB in Ethiopia including the central region where this study was carried out (as the author themselves indicated). So, what new insights are the author intending to reveal to persuade the readers the novelty of this work other than imitating what have been done previously? It would have been more appropriate to apportion the resource used to conduct this study toward pilot study for BTB control and prevention.

***Response:*** *We believe there was enough reason to undertake this study (irrespective of stated PLoS ONE policy). Our paper confirms and updates the importance of bTB as a problem in central Ethiopia. The extent of the problem was not quantified contemporaneously to all for specific control strategies. No other study covering the area attempted a one stage cluster sampling, and none sampled more than 100 farms, the most recent survey being in 2008/9. In the revised version we have also demonstrated differences in within herd prevalence of bTB (stratified by herd size), important information for future design of control strategies. In addition, we have improved understanding of the risk factors among dairy farms in Ethiopia*.

There seems to be a− hierarchical (multi-level) structure of data (study area>>herd>>>individual animals. The use of GLMM to account for infection clustering at various levels is, therefore, appropriate. However, it is recommended that the authors use mathematical expression to help readers identify which model was used (linear regression, logistic regression, and Poisson regression?). Understandably, logistic model is fitting since the outcome variable is of binary type. Nevertheless, this has to be explained to the readers explicitly.

***Response:*** *Accepted. And mathematical expression is included in the revised paper. In the revised paper, detailed description is provided in the statistical analysis section.*

Not so sure why the authors− chose to ignore the use of univariable analysis first to assess effects of individual variables on outcome variable. Understandably, relatively fewer variables were recorded in the study but still plausible to assess their individual and interactive effects on outcomevariable.

***Response:*** *Univariable analysis is included in the revised paper (Table 4). Our large data set allowed more (16) potential risk factors to be considered. We have analyzed by including additional risk factors.*

− Line 80 “Six study areas were selected in the urban areas of central Ethiopia”. The authors did not mention how these areas were selected. Was it random or systematic/purposeful?

***Response:*** *The selection of these study areas was purposeful. The study areas are currently the main milk suppliers for people in Addis Ababa and the surrounding peri-urban areas. This description is included in the revised paper.*

6. PLOS authors have the option to publish the peer review history of their article ([what does this mean?](https://journals.plos.org/plosone/s/editorial-and-peer-review-process#loc-peer-review-history)). If published, this will include your full peer review and any attached files.
If you choose “no”, your identity will remain anonymous but your review may still be made public.
***Response***: *May be we do not understand it well during online submission. We choose "Yes" for the revised paper.*
[NOTE: If reviewer comments were submitted as an attachment file, they will be attached to this email and accessible via the submission site. Please log into your account, locate the manuscript record, and check for the action link "View Attachments". If this link does not appear, there are no attachment files to be viewed.]

- - - - -
For journal use only: PONEDEC3
